# Effects of Pre-Natal Vitamin D Supplementation with Partial Correction of Vitamin D Deficiency on Early Life Healthcare Utilisation: A Randomised Controlled Trial

**DOI:** 10.1371/journal.pone.0145303

**Published:** 2015-12-23

**Authors:** Megan Griffiths, Stephen Goldring, Chris Griffiths, Seif O. Shaheen, Adrian Martineau, Louise Cross, Stephen Robinson, John O. Warner, Angela Devine, Robert J. Boyle

**Affiliations:** 1 Department of Paediatrics, Imperial College London, London, United Kingdom; 2 Centre for Primary Care and Public Health, Blizard Institute, Queen Mary University of London, London, United Kingdom; 3 Department of Endocrinology, Imperial College London, London, United Kingdom; University of Missouri, UNITED STATES

## Abstract

**Background:**

Some observational studies have suggested that higher prenatal Vitamin D intake may be associated with improved health outcomes in childhood. However there have been mixed results in this area with some negative studies, especially for effects on atopic and respiratory outcomes. We examined the effect of prenatal Vitamin D on healthcare utilisation in the first three years of life.

**Methods:**

In an ethnically stratified randomised controlled trial conducted at St Mary’s Hospital London, 180 women at 27 weeks gestation were allocated to no Vitamin D, 800 IU ergocalciferol daily until delivery, or a single oral bolus of 200,000 IU cholecalciferol. Participants were randomised in blocks of 15 using computer-generated numbers and investigators were blinded to group assignment. Supplementation increased maternal and cord blood 25(OH) vitamin D concentrations, but levels remained lower than current recommendations. Primary health economic outcome was overall cost of unscheduled healthcare utilisation in the first three years of life as documented in the child’s electronic health record. Secondary outcomes included cost attributable to: primary and secondary healthcare visits, respiratory and atopic complaints, cost in years 1, 2 and 3 of life and cost and frequency of prescribed medication. All costs were calculated as pounds sterling. Differences between groups were analysed using unpaired t-test or Mann-Whitney U test, and analysis of variance for adjusted analyses.

**Results:**

We assessed 99/180 (55%) complete electronic health records, control (n = 31), daily (n = 36) and bolus (n = 32). We found no difference in total healthcare utilisation costs between the control and daily (mean difference in costs in pounds sterling 1.02, 95%CI -1.60, 1.65; adjusted 1.07, 95%CI -1.62, 1.86) or control and bolus groups (mean difference -1.58, 95%CI -2.63, 1.06; adjusted –1.40, 95%CI -2.45, 1.24). There were no adverse effects of supplementation reported during the trial.

**Conclusions:**

We found no evidence that prenatal vitamin D supplementation from 27 weeks gestation to delivery, at doses which failed to completely correct maternal vitamin D deficiency, influence overall healthcare utilisation in children in the first 3 years.

**Trial Registration:**

Controlled-Trials.com ISRCTN68645785

## Introduction

Hypovitaminosis D is an increasing problem in many regions, where sedentary, indoor lifestyles are thought to be a major contributing factor [1]. There is mixed observational evidence with regards to the effect of pre-natal 25(OH) vitamin D status on child health outcomes. Several studies have linked low vitamin D status to a number of resource intensive childhood health problems including allergy and infection [2–7], in addition to the well-established effects on bone metabolism. However, these links remain controversial with other studies reporting no association [8–11]. Allergy alone accounts for 6% of primary care consultations and 70,000 hospital admissions a year in the UK [12] and the average pre-school UK child contracts 6–8 upper respiratory tract infections (URTIs) annually. Vitamin D supplementation during critical periods of immune development may represent a safe [13] and relatively inexpensive public health intervention to reduce this burden. Thus far there has been a lack of randomised controlled trial evidence to definitively confirm or refute the relationship between prenatal vitamin D and childhood health outcomes found in observational studies. Data published in 2013 from this trial, looking primarily at respiratory outcomes, found no difference between treatment and control arms with regards to a primary outcome of wheeze [14].

In the United Kingdom, all children registered with a primary care practice have an electronic health record (e-HR). This is a contemporaneous record of all health care utilisation from that practice. Use of primary care out of hours services (OOH) or secondary healthcare is also recorded, and the e-HR follows the child if they move so that the e-HR forms a complete record of a child’s healthcare utilisation from birth. Determinants of a child’s overall healthcare utilisation are their underlying health status [15–19], parent mental health and family functioning [20–22], and socioeconomic predictors (age and ethnicity of the child, family income, area of residence, parental educational attainment and social class) [15, 19, 23–25], although not all studies have found an effect of social class [16, 17].

We hypothesized that prenatal vitamin D supplementation during pregnancy would reduce a child’s healthcare utilisation during the first three years of life through improved health status, by using the e-HR to quantify our outcome total healthcare utilisation.

## Methods

### Study Setting and Subjects

This was a follow up study of the offspring of 180 women who took part in an ethnically stratified, parallel group, randomised controlled trial of vitamin D supplementation in pregnancy at St Mary’s Hospital London, a university-affiliated hospital antenatal clinic, between April and November 2007. Eligible participants were women presenting at 27 weeks gestation for routine glucose challenge test from the following ethnic groups: Indian Asian, Middle Eastern, Afro-Caribbean and Caucasian. Participants were excluded if there were severe congenital or developmental abnormalities likely to significantly affect respiratory health or lung function e.g. thoracic dystrophy. There were no withdrawal criteria for this study.

### Ethics Statement

St Mary’s Hospital Research Ethics Committee approved the follow-up study (10/H0712/13). Consent was obtained in person or over the telephone, in which case verbal consent was taken and documented by two separate members of the research team. The ethics committee approved verbal consenting in this manner. The confidentiality of all participants has been preserved under the Data Protection Act.

### Study Design

**Fig 1:**
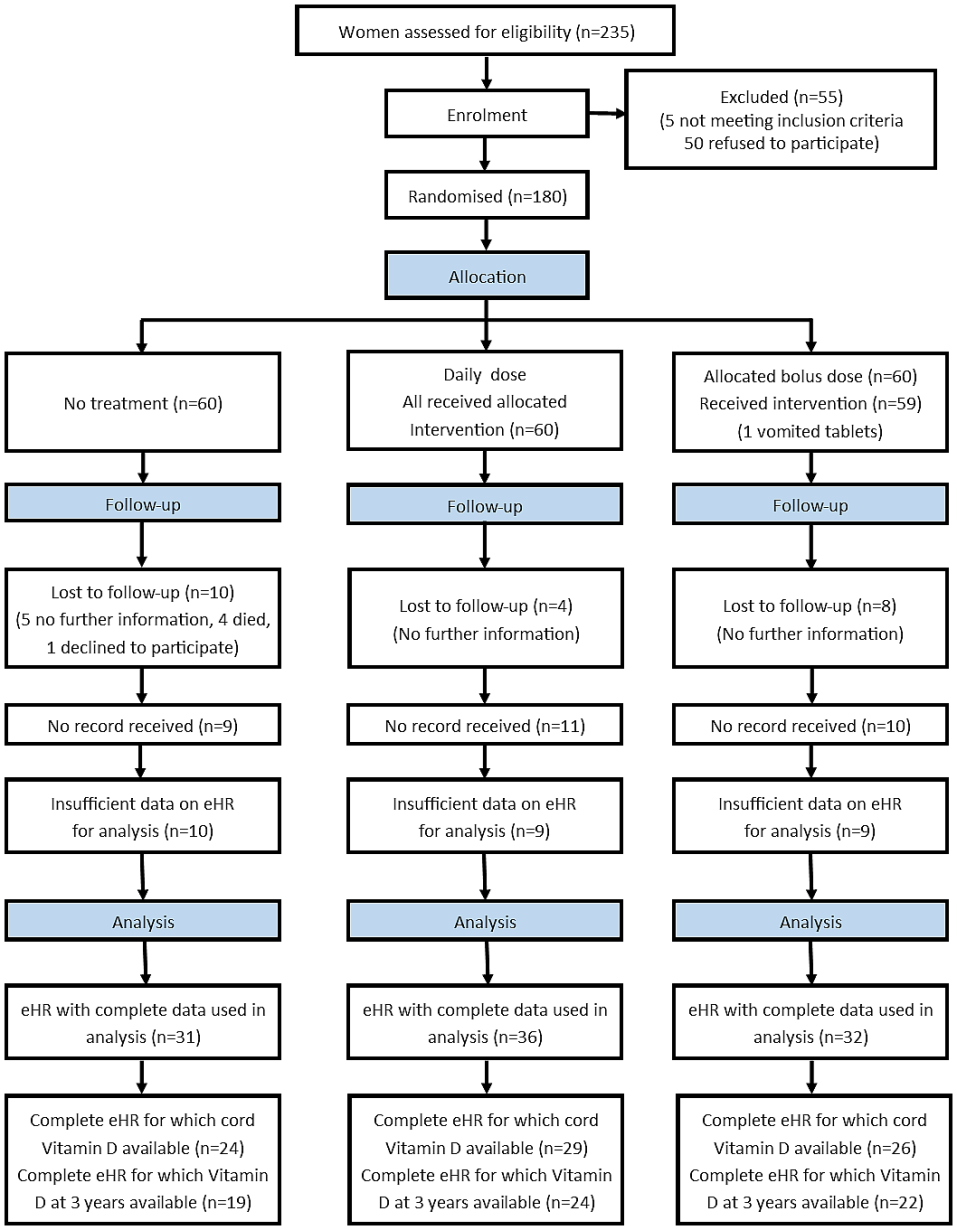
CONSORT flow diagram.

Women were randomised at 27 weeks gestation to no treatment (control), 800 IU ergocalciferol until delivery (daily) or a single oral dose of 200,000 IU cholecalciferol (bolus). The randomisation sequence was generated by an independent person using computer generated random number lists in blocks of 15, stratified by ethnicity in a 1:1:1:1 ratio. The researcher gave participants a study number on entry to the trial, and treatment was allocated from the hospital pharmacy using the randomization list. Women were given instructions to swallow the tablets whole and to avoid other multivitamin supplements containing vitamin D. This trial was conducted before national guidance on routinely providing advice on vitamin D intake during pregnancy was introduced in March 2008 [26]. No adverse effects were reported. Vitamin D deficiency was defined as 25(OH)D <25nmol/L, and insufficiency as 25(OH)D ≥25 but <50nmol/L [27]. We obtained e-HR for those who consented. For analysis, two assessors independently examined the first three e-HRs (84 consultations). The kappa score for their assessment of total healthcare utilisation costs was 0.972, showing excellent agreement. The remaining records were all analysed by a single assessor. All assessors were blinded to the randomisation of participants, with the use of separate trial numbers, until the evaluation of all records was complete. A complete record was defined as having data for at least 11 months out of 12 for each year, and including all three years since birth. The flow of participants is shown in Fig 1.

### Primary and secondary outcomes

The primary outcome of the trial was 25(OH) vitamin D level at birth, and has been reported elsewhere [28]. The primary outcome for the follow-up study was wheeze in the first 3 years of life—this together with other clinical, physiological and immune outcomes has already been reported [14]. The primary outcome of this health economic analysis was the total cost of unscheduled NHS healthcare utilisation across primary and secondary care recorded in the e-HR from birth until the child’s third birthday, including both visit costs and prescription costs. Scheduled care such as routine developmental checks and vaccinations was not included. Secondary outcomes were as follows: total costs for the first, second and third year of life separately; total primary care visit costs, total secondary care visit costs, respiratory costs i.e. healthcare utilisation attributed to wheeze, cough, upper respiratory tract infection, lower respiratory tract infection and bronchiolitis; food allergy costs, eczema costs; prescription costs attributable to wheezing and eczema (separately).

Primary healthcare consultations were classified according to the professional providing the service (general practitioner, out of hours service or nurse) and location (surgery, telephone or home visit). Entries without details were classed as ‘primary care; unknown’. Secondary healthcare utilization was classified as new outpatient appointment, follow-up outpatient appointment, accident and emergency attendance, ward admission or intensive care unit admission. For admissions, the number of nights in hospital was recorded. Entries regarding administrative tasks were not included. Coding definitions are shown in Table 1.

**Table 1:** Diagnostic codes for electronic health records.

Outcome	Definition. Any record of the following in the e-HR
Wheeze	Wheeze or asthma excluding wheeze in the context of bronchiolitis
Cough	Cough excluding cough in the context of URTI or LRTI
URTI	URTI, pharyngitis, rhinorrhoea, laryngitis, tonsillitis, sinusitis or otitis media. Or at least two of fever, sore throat, earache, sticky eye or cough
LRTI	LRTI, chest infection, respiratory infection, bronchitis or pneumonia. Or cough with a record of signs of infection such as crackles on auscultation
Bronchiolitis	Bronchiolitis
Food allergy	Doctor diagnosed food allergy
Eczema	Eczema or eczematous skin

URTI (Upper respiratory tract infection), LRTI (Lower respiratory tract infection).

### Visit costs

Costs were calculated according to standard NHS tariffs for 2009 in pounds sterling, that included training and, where applicable, travel costs and direct care staff costs. All costs are reported in pounds sterling, either as raw or natural log transformed figures. As no unit cost was given for telephone calls with a practice nurse, this was estimated as follows: a telephone consultation with an advanced nurse is six minutes long on average. When applied to the hourly rate for client consultation with a GP practice nurse (£34) this gives an average cost of £3.40 per call. A factor of 1.44 was applied to in hours GP utilization to give the estimated out of hours cost. This is based on the mean cost per hour for out of hours GP services being £82.03 (Inflation adjusted average), [29] which is 44% more than the mean cost per hour for in hours GP services of £57.03 [30]. For secondary care new and follow-up outpatient attendances, a weighted mean of the cost of all types of paediatric referrals for consultant led outpatient care was taken, giving an overall mean of £198.60 for a first appointment and £155.36 for a follow-up appointment. Similar methods were used for accident and emergency attendances, such that the weighted mean for an A&E attendance leading to admission was £129.11, and an A&E attendance without admission £94.93. This cost excludes the use of minor injury services and walk-in centres. The details of NHS costing codes used in analysis can be found in Table 2 [31].

**Table 2:** Costs used for calculation of healthcare utilization, and their source.

Type of Utilization	Unit Cost (GBP)	Source
Primary Care		
Nurse In Surgery	11	Units costs by Curtis 2009 [30]
Nurse Telephone Consultation	3.4	Units costs by Curtis 2009 [30]
Nurse Home Visit	20	Units costs by Curtis 2009 [30]
GP In Surgery	35	Units costs by Curtis 2009 [30]
GP Telephone Consultation	21	Units costs by Curtis 2009 [30]
GP Home Visit	117	Units costs by Curtis 2009 [30]
OOH In Surgery	50.4	Units costs by Curtis 2009 [30] Unit costs by Curtis 2010 [32], NAO report OOH Care [33]
OOH Telephone Consultation	30.24	Units costs by Curtis 2009 [30] Unit costs by Curtis 2010[32] NAO report OOH Care[33]
OOH Home Visit	168.48	Units costs by Curtis 2009 [30]Unit costs by Curtis 2010 [32], NAO report OOH Care [33]
Secondary Care		
First Attendance to Consultant Led OP	198.6	Appendix NSRC4 NHS Trust [31]
Follow-up Attendance to Consultant Led OP	155.36	Appendix NSRC4 NHS Trust [31]
A &E Attendance Leading to Admission	129.11	Appendix NSRC4 NHS Trust [31]
A&E Attendance Not Leading to Admission	94.93	Appendix NSRC4 NHS Trust [31]
Inpatient Stays		
Kidney or Urinary Tract Infections with length of stay less 1 day or less	392	Appendix NSRC4 NHS Trust Non-Elective Inpatient (Short Stay) Code: LA04G [31]
Penile Conditions and Minor Procedures 18 years and under	1307	Appendix NSRC4 NHS Trust Elective Inpatient Code:LB32C [31]
Major Neonatal Diagnoses	2075	Appendix NSRC4 NHS Trust Non-Elective Inpatient (Long Stay) Code: PB01Z [31]
Major Congenital Conditions under 1 year without CC	537	Appendix NSRC4 NHS Trust Non-Elective Inpatient (Short Stay) Code: PA59D [31]
Major Congenital Conditions 1 year and over without CC	535	Appendix NSRC4 NHS Trust Non-Elective Inpatient (Short Stay) Code: PA59F [31]
Acute Febrile illness length of stay 4 days or less without CC	378	Appendix NSRC4 NHS Trust Non-Elective Inpatient (Short Stay) Code: WA04U [31]
Viral Infections with length of stay 2 day or more	922	Appendix NSRC4 NHS Trust Non-Elective Inpatient (Short Stay) Code: PA19B [31]
Fever unspecified without CC	487	Appendix NSRC4 NHS Trust Non-Elective Inpatient (Short Stay) Code: PA20B [31]
Other Viral illness without CC	368	Appendix NSRC4 NHS Trust Non-Elective Inpatient (Short Stay) Code: WA06Y [31]
Feeding Difficulties and Vomiting without CC	455	Appendix NSRC4 NHS Trust Non-Elective Inpatient (Short Stay) Code: PA28B [31]
Infectious and Non-Infectious Gastroenteritis without CC	459	Appendix NSRC4 NHS Trust Non-Elective Inpatient (Short Stay) Code: PA21B [31]
Intermediate Mouth or Throat Procedures 18 years and under with CC	1799	Appendix NSRC4 NHS Trust Elective Inpatient Code: CZ02S [31]
Other Respiratory Diagnoses without CC	342	Appendix NSRC4 NHS Trust Non-Elective Inpatient (Short Stay) Code: DZ19C [31]
Acute Bronchiolitis without CC	489	Appendix NSRC4 NHS Trust Non-Elective Inpatient (Short Stay) Code: PA15B [31]
Acute Bronchiolitis with CC	573	Appendix NSRC4 NHS Trust Non-Elective Inpatient (Short Stay) Code:PA15A [31]
Lower Respiratory Tract Disorders without Acute Bronchiolitis with length of stay 1 day or more without CC	544	Appendix NSRC4 NHS Trust Non-Elective Inpatient (Short Stay) Code: PA14D [31]
Minor Skin disorders category 2 without CC	396	Appendix NSRC4 NHS Trust Non-Elective Inpatient (Short Stay) Code: JD05C [31]
Non-Malignant General Abdominal Disorders with length of stay 0 days	410	Appendix NSRC4 NHS Trust Non-Elective Inpatient (Short Stay) Code: FZ47C [31]

GP- general practitioner, OOH—out of hours, OP- outpatient, CC- critical care.

### Prescription costs

Prescription costs were calculated from the online version of the British National Formulary for Children 2011 [34]. These reflect the cost of drugs issued and not the cost of administration associated with delivery to patients. The price of the pharmaceutical brand specified in the record was taken. If no brand was specified the cheapest form of that medication with the same mode of delivery was used. Where costs were itemized as a net price per measured unit of drug (for example per 100ml), the price was applied proportional to the amount prescribed. Where the drug was priced per set volume (for example per tube in the case of creams) then the number of tubes required to cover the volume prescribed was calculated. The summation of the medication costs in each record was then subjected to deflation calculations using inflation indices [32]. As no inflation indices for 2010/11 were available at the time of calculation, the indices to deflate 2010 to 2009 were used twice, thereby assuming a similar rate of inflation across that time. Deflating prices to 2008/09 brought them to the same base year as the healthcare utilization costs. The price of Anusol cream and Dentinox oral solution were not available within the BNFC 2010 and were priced from Boots pharmacy; each of these were prescribed once. Whether or not a child was prescribed drugs relevant to eczema (emollient creams and bath oils, topical corticosteroids with or without antibiotics or antiseptic and calcineurin inhibitors), asthma (inhaled corticosteroids, B2 agonists, leukotriene inhibitors and delivery devices) or food allergy (adrenaline autoinjectors and antihistamines) was separately recorded.

### Statistical analyses

A database was assembled using Microsoft Excel. Data were explored using histograms and the following became normally distributed after natural log transformation: total healthcare utilization, total visit costs, total prescription costs, total primary care costs, total year 1 costs and overall respiratory costs. Levels of 25(OH)D at baseline in the mother, in cord blood and at age three years in the child were all normally distributed after natural log transformation. 0.1 was added to all zero values to allow natural log transformation. The following data remained non-normally distributed despite natural log transformation: total secondary care costs, year 2 and 3 costs, respiratory costs, food allergy costs, eczema costs and prescription costs for eczema and wheezing medications.

Primary analysis evaluated control versus daily supplement and control versus bolus supplement. Secondary analysis included bolus and daily vitamin D groups combined (combined vitamin D) compared with the control group and the relationship between 25(OH)D levels measured in cord blood at delivery and in the child at age three years. Differences between groups were analysed using the independent student t-test or Mann-Whitney U test according to whether the data were normally distributed or not. Where analysis was performed on log transformed data, the results have undergone exponentiation for presentation in the tables. Analysis of variance was performed on normally distributed data using potential covariates that have been associated with healthcare utilisation in the literature including mother’s age, mother’s age on leaving full time education, gestational age, ethnicity, parity, maternal body mass index (BMI), smoking during pregnancy and mode of delivery [24]. Logistic regression was used to calculate adjusted odds ratios for dichotomised data regarding prescriptions for wheeze and eczema related drugs. All analyses were performed using SPSS version 19.0 (IBM, Chicago USA). A value of p<0.05 was considered statistically significant.

Formal Bonferroni correction for multiple analyses was not performed, but statistical significance was interpreted cautiously where multiple analyses had been made [35].

## Results

Of the 180 offspring from the original trial, we successfully obtained 130 e-HRs from their GP’s between January and May 2011. 31 had incomplete data and could not be used, leaving 99/180 (55%) infants with complete e-HR data for analysis. An additional 12 records were incomplete but had at least one year’s health data for analysis. Incomplete records were due to inadequate GP summaries or where the patient had moved GP practice over the last three years and we were unable to obtain the old or new records. Only records with complete data were used for primary analysis. Participant flow through the study can be found in Fig 1. Table 3 shows the characteristics of children for whom complete e-HR data was obtained and analysed and those who were not analysed (either incomplete or not received). There were minor differences between groups with a slightly higher proportion of Middle Eastern children with incomplete records and a slightly higher proportion of Black children for whom no record was received. However, the groups remain relatively similar in most respects. The characteristics of children with complete records in each randomization group are shown in Table 4.

**Table 3:** Characteristics of children with complete e-HR data, incomplete e-HR data and for whom no e-HR data were received.

	Complete(n = 99)	Incomplete(n = 12)	Not Received(n = 69)
Male sex, n (%)	52/99 (53)	7/12 (58)	34/69 (49)
Gestational age, weeks	39.6 (1.57)	40.5 (1.27)	39.6 (1.27)
Delivery by caesarean section, n (%)	30/99 (30)	4/12 (33)	23/69 (33)
Cord blood 25(OH) Vitamin D available, n (%)	79/99 (80)	7/12 (58)	55/69 (80)
Mean cord blood 25(OH) Vit D (nmol/L)	26.9 (13.6)	30.6 (19.1)	24.3 (11.0)
Maternal Ethnicity, n (%)			
Asian	25/99 (25)	3/12 (25)	17/69 (25)
Middle Eastern	25/99 (25)	5/12 (42)	15/69 (22)
Black	21/99 (21)	2/12 (17)	21/69 (30)
Caucasian	28/99 (28)	2/12 (17)	16/69 (23)
Maternal age at booking, years	32 (6.45)	30 (5.47)	30 (6.02)
Maternal smoking in pregnancy, n (%)	13/99 (13)	0/12 (0)	6/69 (9)
Multiparous, n (%)	65/99 (66)	5/12 (42)	31/69 (45)
Maternal BMI at booking	27 (6.07)	26 (5.51)	25 (4.99)
Maternal 25(OH) Vit D at delivery available n(%)	86/99 (87)	10/12 (83)	62/69 (90)
Maternal 25(OH) Vit D at delivery	42.65 (22.2)	44.0 (22.3)	37.9 (18.0)

Continuous data are presented as mean (standard deviation). BMI = body mass index.

**Table 4:** Characteristics of children with complete e-HR data.

	Control (n = 31)	Daily (n = 36)	Bolus (n = 32)
Male sex, n (%)	15/31 (48)	22/36 (61)	17/32 (53)
Gestational age, months	39.92 (1.28)	39.40 (1.53)	39.72 (1.66)
Delivery by caesarean section, n (%)	10/31 (32)	10/36 (28)	10/32 (31)
Cord blood 25(OH) Vitamin D available, n (%)	24/31 (77)	29/36 (81)	26/32 (81)
Mean cord blood 25(OH) Vit D (nmol/L)	18.8 (9.4)	34.1 (16.3)	26.4(8.31)
Maternal Ethnicity, n (%)			
Asian	6/31 (19)	10/36 (28)	9/32 (28)
Middle Eastern	11/31 (35)	8/36 (22)	6/32 (19)
Black	5/31 (16)	8/36 (22)	8/32 (25)
Caucasian	9/31 (29)	10/36 (28)	9/32 (28)
Maternal age at booking, years	30 (7)	32 (6)	32 (6)
Maternal highest education, years	20 (3)	20 (4)	20 (3)
Family history of atopy, n (%)	13/29 (45)	23/35 (63)	15/31 (48)
Maternal smoking in pregnancy, n (%)	5/31 (16)	5/36 (14)	3/31 (10)
Number children in household	2 (1)	2 (1)	3 (1)
Multiparous, n (%)	19/31 (61)	25/36 (69)	21/32 (66)
Maternal BMI > 30 at booking, n (%)	9/31 (29)	4/36 (11)	4/32 (12.5)
Maternal 25(OH) Vit D at delivery available n(%)	26/31(83)	33/36 (92)	27/32 (84)
Maternal 25(OH) Vit D at delivery	33.0 (17.6)	55.3(27.0)	36.5(9.5)

Continuous and ordinal data are presented as mean (standard deviation). BMI = body mass index.

### Effect of prenatal vitamin D supplementation on healthcare utilisation

**Fig 2:**
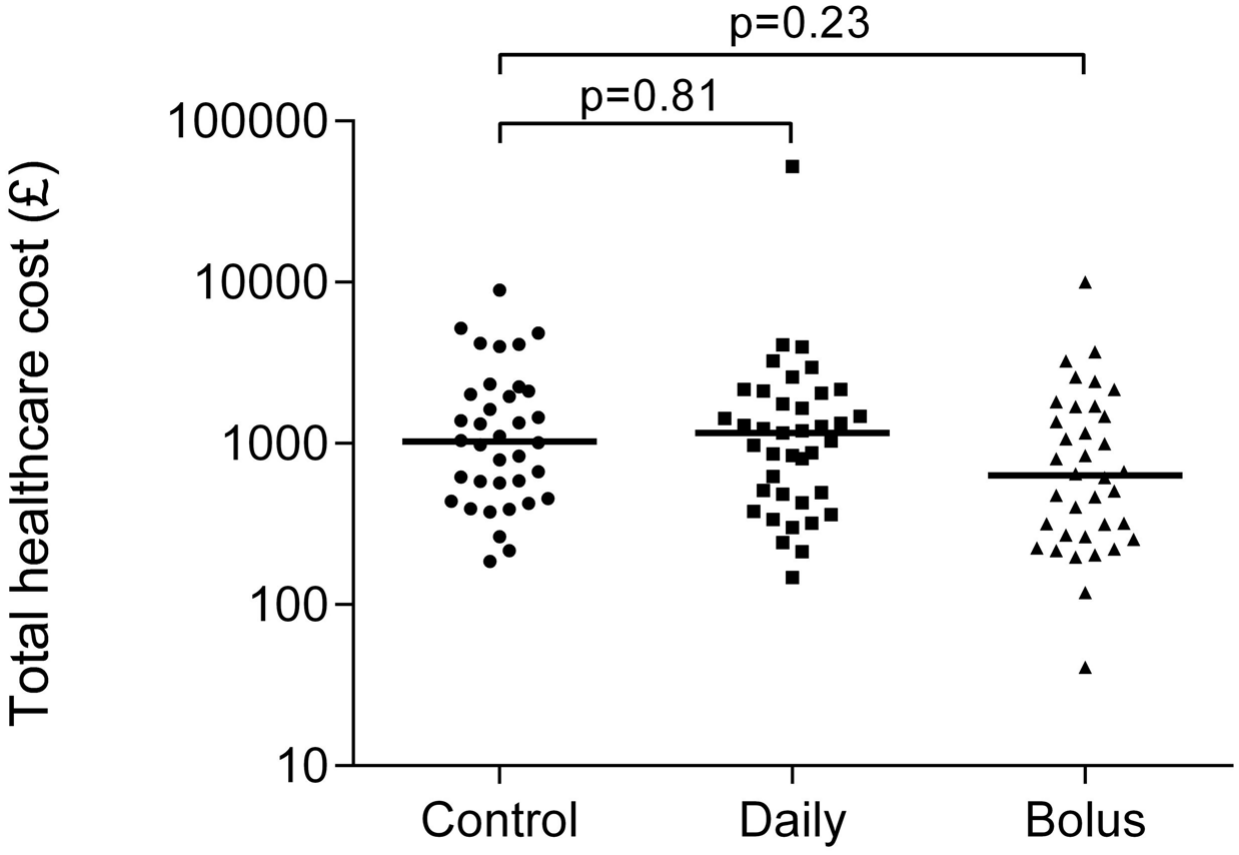
Effect of prenatal vitamin D randomisation on total healthcare utilisation in the first three years of life. Horizontal bars represent medians and p values are for adjusted analyses.

There was no significant difference in the primary outcome measure, total unscheduled healthcare utilisation, between treatment groups (Fig 2). Mean difference between control and daily vitamin D supplementation in Ln transformed total costs was—0.02 (95%CI -0.47, 0.50); adjusted mean difference 0.07 (95%CI -0.48, 0.62). For bolus vitamin D supplementation versus controls the mean difference was -0.46 (95%CI-0.97, 0.06); adjusted mean difference -0.34 (95%CI -0.90, 0.22).

We also found no significant difference in secondary outcomes including total prescription costs, total visit costs, costs in years 1, 2 and 3 and costs specific to respiratory or allergic disorders, between offspring in the daily treatment group compared to controls (Table 5). There was weak evidence that secondary care health utilisation costs (p = 0.048) and year 3 costs (p = 0.045) were reduced in the bolus group (Table 6). When treatment groups were combined for analysis, no difference was found from controls (Table 7).

**Table 5:** Healthcare utilisation in the first three years of life. Daily vitamin D versus control.

	n	Control*	n	Daily*	Mean difference (95% CI)	p	n	Adjusted** mean difference (95% CI)	p
Total cost^†^	31	1176.14 (437.03, 3165.29)	36	1187.97 (441.42, 3197.10)	1.02 (-1.60, 1.65)	0.95	53	1.07 (-1.62, 1.86)	0.81
All Visits^†^	31	1130.03 (419.90, 3041.18)	36	1118.79 (407.48, 3071.74)	-1.01 (-1.65, 1.60)	0.95	53	1.02 (-1.70, 1.79)	0.93
Prescription cost^†^	31	25.28 (4.06, 157.60)	36	35.52 (11.59, 108.85)	1.40 (-1.49, 2.94)	0.36	53	1.65 (-1.43, 3.90)	0.25
Year 1 cost^†^	31	287.15 (38.09, 2164.62)	36	383.75 (86.49, 1702.75)	1.34 (-1.75, 3.16)	0.50	53	1.67 (-1.58, 4.39)	0.30
Year 2 cost	31	247 (103, 607)	36	248 (123, 487)	-	0.92	-	-	-
Year 3 cost	31	239 (103, 552)	36	212 (96, 463)	-	0.57	-	-	-
Primary care cost^†^	31	387.61 (145.47, 1032.77)	36	450.39 (242.26, 837.15)	1.15 (-1.28, 1.72)	0.47	53	1.34 (-1.16, 2.05)	0.20
Secondary care cost	31	632 (206, 1853)	36	368 (155, 1502)	-	0.42	-	-	-
Wheeze cost	31	0 (0, 0)	36	0 (0, 0)	-	0.71	-	-	-
Cough cost	31	0 (0, 36)	36	0 (0, 36)	-	0.26	-	-	-
URTI cost	31	180 (36, 324)	36	150 (54, 234)	-	0.78	-	-	-
LRTI cost	31	0 (0, 0)	36	0 (0, 0)	-	0.75	-	-	-
Bronchiolitis cost	31	0 (0, 0)	36	0 (0, 0)	-	0.89	-	-	-
Overall resp cost^†^	31	170.72 (27.39, 1064.22)	36	141.17 (27.11, 735.10)	-1.21 (-3.16, 2.14)	0.69	-	-	-
Food allergy cost	31	0 (0, 0)	36	0 (0, 0)	-	0.35	-	-	-
Eczema cost	31	0 (0, 88)	36	0 (0, 36)	-	0.62	-	-	-

URTI (Upper respiratory tract infection), LRTI (Lower respiratory tract infection).

All costs are in pounds sterling.

*Mean ± 95% CI or Median (IQR)

**Adjusted for mother’s age, mother’s age on leaving education and gestational age as potential covariates, and ethnicity (Caucasian, Asian, Middle Eastern, Black), parity, BMI>30, smoking during pregnancy, mode of delivery (vaginal, instrumental or caesarean) as potential cofactors.

^†^Analysis performed on Ln data, results have undergone exponentiation for presentation in tables.

**Table 6:** Healthcare utilization in the first three years of life. Bolus vitamin D versus control.

	n	Control*	n	Bolus*	Mean difference (95% CI)	p	n	Adjusted** mean difference (95% CI)	p
Total cost^†^	31	1176.14 (437.03, 3165.29)	31	742.48 (257.24, 2413.08)	-1.58 (-2.63, 1.06)	0.08	49	-1.40 (-2.45, 1.24)	0.23
All visit costs^†^	31	1130.03 (419.90, 3041.18)	32	706.27 (239.85, 2079.74)	1.60 (-2.69, 1.05)	0.08	49	-1.43 (-2.53, 1.23)	0.21
All prescription cost^†^	31	25.28 (4.06, 157.60)	32	22.42 (4.85, 103.54)	1.12 (-2.61, 2.08)	0.79	49	1.23 (-1.95, 3.00)	0.63
Year 1 cost^†^	31	287.15 (38.09, 2164.62)	32	223.63 (62.80, 796.32)	1.28 (-2.97, 1.82)	0.56	49	1.02 (-2.36, 2.46)	0.96
Year 2 cost	31	247 (103, 607)	32	202 (101, 603)	-	0.75	-	-	-
Year 3 cost	31	239 (103, 552)	32	142 (36, 333)	-	0.04	-	-	-
Primary care cost^†^	31	387.61 (145.47, 1032.77)	32	383.75 (169.02, 871.32)	1.02 (-1.60, 1.55)	0.95	49	1.01 (-1.57, 1.60)	0.98
Secondary care cost	31	632 (206, 1853)	32	313 (0, 848)	-	0.04	-	-	-
Wheeze visit cost	31	0 (0, 0)	32	0 (0, 0)	-	0.22	-	-	-
Cough visit cost	31	0 (0, 36)	32	0 (0, 36)	-	0.59	-	-	-
URTI visit cost	31	180 (36, 324)	32	108 (72, 255)	-	0.91	-	-	-
LRTI visit cost	31	0 (0, 0)	32	0 (0, 36)	-	0.31	-	-	-
Bronchiolitis visit cost	31	0 (0, 0)	32	0 (0, 0)	-	0.59	-	-	-
Overall resp cost^†^	31	170.72 (27.39, 1064.22)	32	137.00 (15.96, 1176.15)	-1.25 (-3.42, 2.20)	0.66	-	-	-
Food allergy cost	31	0 (0, 0)	32	0 (0, 0)	-	1.00	-	-	-
Eczema cost	31	0 (0, 88)	32	0 (0, 36)	-	0.77	-	-	-

URTI (Upper respiratory tract infection), LRTI (Lower respiratory tract infection).

All costs are in pounds sterling.

*Mean ± 95% CI or Median (IQR)

**Adjusted for mother’s age, mother’s age on leaving education and gestational age as potential covariates, and ethnicity (Caucasian, Asian, Middle Eastern, Black), parity, BMI>30, smoking during pregnancy, mode of delivery (vaginal, instrumental or caesarean) as potential cofactors.

^†^Analysis performed on Ln data, results have undergone exponentiation for presentation in the table.

**Table 7:** Healthcare utilization costs in the first three years of life. Combined vitamin D versus control.

	n	Control*	n	Combined*	Mean difference	p	n	Adjusted** mean difference	p
Total cost^†^	31	1176.14 (437.03, 3165.29)	68	953.37 (333.62, 2724.39)	-1.23 (-1.92, 1.27)	0.36	84	-1.17 (-1.90, 1.39)	0.52
All visit cost^†^	31	1130.03 (419.90, 3041.18)	68	897.85 (311.06, 2591.52)	-1.27 (-1.95, 1.25)	0.31	84	-1.21 (-1.97, 1.35)	0.45
All prescription cost^†^	31	25.28 (4.06, 157.60)	68	28.50 (7.32, 111.05)	1.14 (-1.70, 2.18)	0.70	84	1.34 (-1.49, 2.66)	0.41
Year 1 total cost^†^	31	287.15 (38.09, 2164.62)	68	298.87 (74.44, 1199.91)	1.04 (-1.93, 2.08)	0.91	84	1.25 (-1.70, 2.63)	0.56
Year 2 total cost	31	247 (103, 607)	68	244 (108, 558)	-	0.81	-	-	-
Year 3 total cost	31	239 (103, 552)	68	168 (60, 430)	-	0.14	-	-	-
Primary care visit cost^†^	31	387.61 (145.47, 1032.77)	68	415.72 (202.35, 854.06)	1.07 (-1.32, 1.52)	0.70	84	1.16 (-1.25, 1.67)	0.42
Secondary care visit cost	31	632 (206, 1853)	68	316 (103, 1050)	-	0.11	-	-	-
Wheeze visit cost	31	0 (0,0)	68	0 (0, 0)	-	0.37	-	-	-
Cough visit cost	31	0 (0, 36)	68	0 (0, 36)	-	0.33	-	-	-
URTI visit cost	31	180 (36, 324)	68	130 (72, 252)	-	0.82	-	-	-
LRTI visit cost	31	0 (0, 0)	68	0 (0, 24)	-	0.68	-	-	-
Bronchiolitis visit cost	31	0 (0, 0)	68	0 (0, 0)	-	0.70	-	-	-
Overall resp cost^†^	31	170.72 (27.39, 1064.22)	68	138.38 (347.23, 1107.65)	-1.23 (2.92, 1.93)	0.64	-	-	-
Food allergy cost	31	0 (0, 0)	68	0 (0, 0)	-	0.50	-	-	-
Eczema cost	31	0 (0, 88)	68	0 (0, 36)	-	0.90	-	-	-

URTI (Upper respiratory tract infection), LRTI (Lower respiratory tract infection).

All costs are in pounds sterling.

*Mean ± 95% CI or Median (IQR)

**Adjusted for mother’s age, mother’s age on leaving education and gestational age as potential covariates, and ethnicity (Caucasian, Asian, Middle Eastern, Black), parity, BMI>30, smoking during pregnancy, mode of delivery (vaginal, instrumental or caesarean) as potential cofactors

^†^Analysis performed on Ln data, results have undergone exponentiation for presentation in table.

### Effect of prenatal vitamin D supplementation on need for prescription medications

Prescribing data were also dichotomized according to whether any prescription was made in the first three years of life. There was weak evidence that offspring of mothers who received bolus vitamin D were more likely to have been prescribed a wheezing medication (p = 0.05), and that offspring of mothers who received daily vitamin D were more likely to have been prescribed an eczema medication (p = 0.04) (Table 8). We therefore evaluated the number of prescriptions for medicated skin creams or wheezing medications, to further explore the possible association with eczema and wheezing medications. Here we found no difference in the number of prescriptions issued for medicated skin creams (median 1.0 IQR 0.0, 2.0 combined vitamin D versus 0.0 (0.0, 2.0) controls; p = 0.27) or wheezing medications (median 0.0 IQR 0.0, 1.0 combined vitamin D versus 0.0 (0.0, 0.0) controls; p = 0.42). There were insufficient data to analyse bolus and daily vitamin D groups separately for this outcome.

**Table 8:** Any prescription for wheezing or eczema. Daily, bolus and combined groups vs controls.

	Control n (%)	Treatment group, n (%)	Unadjusted OR (95%CI)	p	n	aOR^1^ (95% CI)	p
Wheezing medications							
Daily vs controls	4/31 (12.9)	8/36 (22.2)	1.93 (0.66, 1.97)	0.32	66	3.19 (0.57, 17.77)	0.19
Bolus vs controls	4/31 (12.9)	11/32 (34.4)	3.54 (0.98, 12.70)	0.05	62	4.07 (0.98, 16.84)	0.05
Combined vs controls	4/31 (12.9)	19/68 (27.9)	2.62 (0.81, 8.49)	0.11	96	3.08 (0.85, 11.24)	0.09
Eczema medications							
Daily vs controls	14/31 (45.2)	22/36 (61.1)	1.91 (0.72, 5.06)	0.19	66	3.60 (1.05, 12.34)	0.04
Bolus vs controls	14/31 (45.2)	15/32 (46.9)	1.07 (0.40, 2.89)	0.89	62	2.13 (0.60, 7.53)	0.24
Combined vs controls	14/31 (45.2)	37/68 (54.4)	1.45 (0.62, 3.40)	0.39	96	2.22 (0.82, 6.02)	0.12

aOR (Adjusted odds ratio).

^1^Adjusted for mother’s age, mother’s age on leaving education and gestational age as potential covariates, and ethnicity (Caucasian, Asian, Middle Eastern, Black), parity, BMI>30, smoking during pregnancy and mode of delivery (vaginal, instrumental or caesarean) as potential cofactors.

### Association between prenatal or postnatal vitamin D level and healthcare utilisation

**Fig 3:**
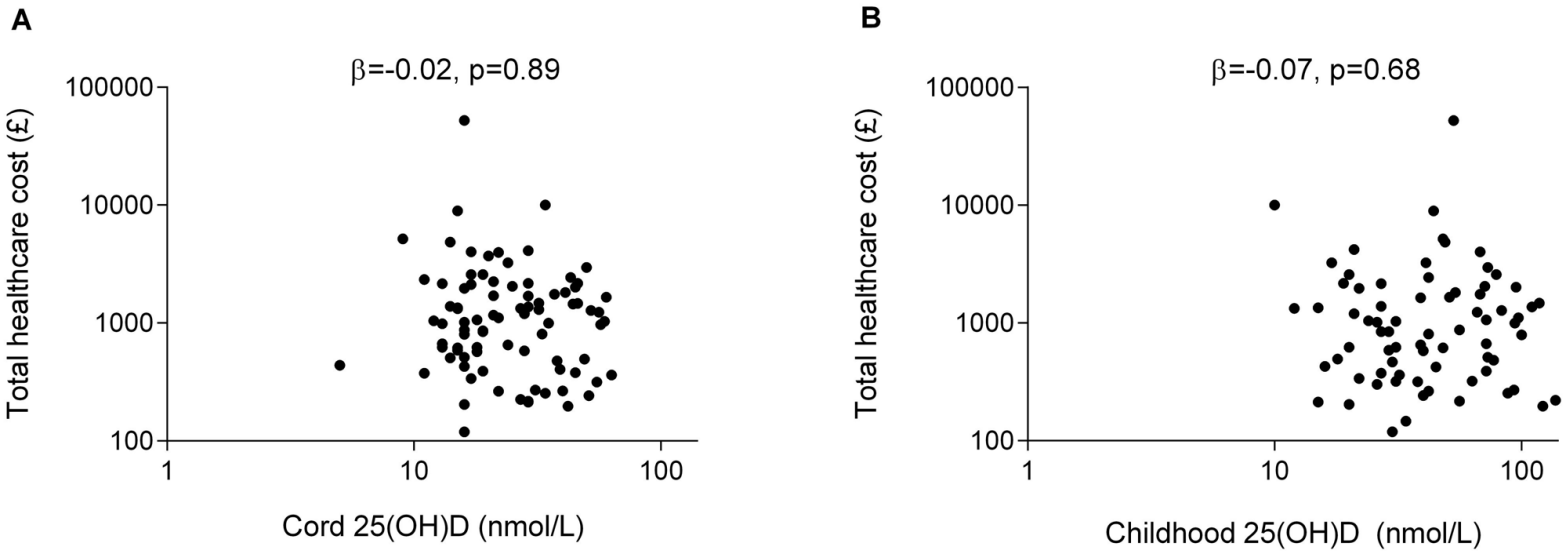
Cord and childhood 25(OH)D levels and total healthcare utilisation in the first three years of life.Adjusted correlation (Beta coefficient, β) between 25(OH) vitamin D levels measured at A) Birth and total healthcare utilization at age three years B) Age three years and total health care utilization by age three years.

Of the 99 offspring with complete e-HR data, cord levels of 25(OH)D were available for 79, and 25(OH)D levels at age three years for 65. There was no correlation between either of these measures and any healthcare utilisation outcome (Fig 3A and 3B; Table 9; Table 10). To further investigate the weak evidence for a link between cord blood 25(OH) vitamin D concentration and eczema cost (Table 9), we analysed whether there was a relationship between cord 25(OH) vitamin D level and prescription of a medicated cream (aOR 2.52, p = 0.10, 95% CI 0.85–7.47) and whether cord 25(OH) vitamin D was associated with the number of eczema medications prescribed (r = 0.07, p = 0.91, 95% CI -0.72–7.80). No evidence for an association was found.

**Table 9:** Cord 25(OH)D levels and healthcare utilisation in the first three years of life.

Outcome	n	p	β or ρ	n	Adjusted^1^ β	p
Ln total healthcare	79	0.58	-0.06	76	-0.02	0.89
Ln all visit cost	79	0.54	-0.07	76	-0.01	0.93
Ln all prescription cost	79	0.78	-0.03	76	0.12	0.36
Ln year 1 cost	79	0.57	-0.07	76	0.01	0.94
Year 2 cost	79	0.76	-0.04	-	-	-
Year 3 cost	79	0.57	0.07	-	-	-
Ln primary care cost	79	0.96	-0.01	76	0.12	0.36
Secondary care cost	79	0.62	-0.06	-	-	-
Wheeze cost	79	0.95	0.01	-	-	-
Cough cost	79	0.98	-0.003	-	-	-
UTRI cost	79	0.42	-0.09	-	-	-
LRTI cost	79	0.28	0.12	-	-	-
Bronchiolitis cost	79	0.55	-0.07	-	-	-
Ln Overall Resp cost	79	0.35	-0.10	76	-0.05	0.74
Food allergy cost	79	0.86	-0.02	-	-	-
Eczema cost	79	0.12	0.18	-	-	-

β (Beta coefficient), ρ (Spearmans correlation coefficient), URTI (Upper respiratory tract infection, LRTI (Lower respiratory tract infection).

^1^Adjusted for treatment group, mother’s age, mother’s age on leaving education and gestational age as potential covariates, and ethnicity (Caucasian, Asian, Middle Eastern, Black), parity, BMI>30, smoking during pregnancy, mode of delivery (vaginal, instrumental or caesarean) as potential cofactors.

**Table 10:** Childhood 25(OH)D levels and healthcare utilisation in the first three years of life.

Outcome	n	p	β or ρ	n	Adjusted^1^ β	p
Ln total healthcare	65	0.52	-0.08	63	-0.07	0.68
Ln all visit cost	65	0.49	-0.09	63	-0.08	0.66
Ln all prescription cost	65	0.88	0.02	63	-0.007	0.97
Ln year 1 cost	65	0.63	-0.06	63	-0.05	0.76
Year 2 cost	65	0.52	-0.08	-	-	-
Year 3 cost	65	0.24	-0.15	-	-	-
Ln primary care cost	65	0.86	0.02	63	0.12	0.93
Secondary care cost	65	0.70	-0.05	-	-	-
Wheeze cost	65	0.72	0.05	-	-	-
Cough cost	65	0.56	0.07	-	-	-
UTRI cost	65	0.21	-0.16	-	-	-
LRTI cost	65	0.50	-0.09	-	-	-
Bronchiolitis cost	65	0.94	-0.01	-	-	-
Ln overall Resp cost	65	0.11	-0.20	63	-0.14	0.45
Food allergy cost	65	-	-	-	-	-
Eczema cost	65	0.86	0.02	-	-	-

β (Beta coefficient), ρ (Spearmans correlation coefficient), URTI (Upper respiratory tract infection, LRTI (Lower respiratory tract infection).

^1^Adjusted for treatment group, mother’s age, mother’s age on leaving education and gestational age as potential covariates, and ethnicity (Caucasian, Asian, Middle Eastern, Black), parity, BMI>30, smoking during pregnancy, mode of delivery (vaginal, instrumental or caesarean) as potential cofactors.

## Discussion

We evaluated whether two specific dosing regimens of prenatal vitamin D supplementation could reduce health care utilisation in the first three years of life. We found no consistent evidence for a treatment effect on overall or disease-specific unscheduled healthcare utilisation in the first three years, although it should be noted that neither of the intervention regimens resulted in adequate vitamin D levels in mothers or cord blood, judged by current international vitamin D recommendations [36, 37]. We found no evidence that prenatal vitamin D status is related to healthcare utilisation, however one might argue that the very small number of subjects with adequate vitamin D status limited the power of this analysis. These data contrast with recent findings that higher dose vitamin D during pregnancy and infancy can reduce upper respiratory tract infections in infancy [38]. Our new findings suggest that prenatal maternal vitamin D levels may be less important than postnatal infant vitamin D levels for determining infant health outcomes, at least within the insufficient/deficient range. Strength is lent to this conclusion when the healthcare utilisation data are contextualised within the other clinical data obtained from this trial in which supplementation did not influence odds of respiratory or allergic symptoms, or measures of lung function/inflammation and immune development [14]. Our data failed to show any significant difference between the total healthcare costs of children from control vs. supplemented mothers, with an adjusted mean difference of £1.17 (p = 0.52). Considering the cost of the pharmaceuticals required for intervention (as per 2011); of £50.18 for daily supplement and £10.59 for bolus dosing, this suggests that supplementation solely for this purpose would not be cost-effective. [39]

Studies in other populations, usually with higher prenatal vitamin D status than ours, have found an inverse relationship between cord blood 25(OH) vitamin D level and child health outcomes [4]. However intervention trials have not yet shown this relationship to be a causal one. The findings from our trial, the first prenatal vitamin D intervention trial to report child health outcomes, suggest that either the dosing regimen used in this study was inadequate, or that the relationship between cord blood 25(OH) vitamin D and child health seen in observational studies may be related to hidden confounders. It is important to consider the generalisability and limitations of these conclusions. First the effect (or lack of effect) of the specific timing and dose of vitamin D used in our study cannot be generalised to other dosing and timing schedules. While our interventions did result in at least 50% higher cord blood 25(OH)D concentrations compared with no treatment, cord blood levels were significantly lower in our intervention groups [daily dose 26nmol/l (IQR 17–45); bolus dose 25nmol/l (IQR 18–34)] than in studies that have found protective associations between higher early life 25(OH)D levels on wheezing or respiratory tract infections in early childhood (Camargo 44nmol/L (IQR 29–78) [4]; Morales 74nmol/L (IQR 56–93) [6]; Belderbos 82nmol/L (SE 3.5) [40]). The recent New Zealand intervention trial showing reduced acute respiratory tract infections between 6 and 18 months of life in offspring of Vitamin D supplemented mothers found a higher vitamin D dose was necessary for clinical efficacy, and infants were supplemented in addition to mothers [38]. Maternal serum 25(OH) vitamin D levels in that trial were higher at randomization, and rose to a higher level in the supplemented groups—from ~60 to ~100 nmol/L; compared to ~26 to ~40 nmol/L in our trial. At the time of the initial trial the national UK recommendation for supplementation during pregnancy was 400IU (10mcg) once daily. This continues to be recommended and is integrated into the ‘Healthy Start’ multivitamin tablets. However, guidance from the National Institute for Clinical Excellence and Royal College of Obstetricians and Gynaecologists in the UK now acknowledge this as a minimum dose for all pregnant or breastfeeding women and that higher dosing should be considered in those at high risk of vitamin D deficiency [41, 42]. More recent guidance from the Institute of Medicine estimates 400IU as average daily requirement in adults in the US and Canada but recommends that supplementation of 600IU would ensure most people achieved sufficiency in their target population [37, 43] In a review by Holick et al. in 2011 greater intervention was suggested with baseline dosing of 600IU, increasing to 1500-2000IU in those at risk of deficiency. In addition, higher thresholds of 25(OH) Vitamin D were recommended for deficiency (<50nmol/l) and insufficiency (52.5–72.5nmol/l) [36]. Despite our daily dosing being above the baseline recommendation and showing increase in maternal and cord blood 25(OH) Vitamin D, by these definitions only around 30% of the women in the trial achieved sufficiency, therefore our data likely reflect outcomes looking at the shift from deficient to insufficient and not full replenishment. With the endorsement of not only different dosing regimes but different safe upper limits (4,000 IU/day (IOM) versus upto 10,000IU/day (Holick et al.), there remains an important question whether prenatal vitamin D supplementation at doses which reliably achieve sufficiency in the target population, can improve healthcare outcomes for young children. The relationship between prenatal vitamin D status and child health outcomes may not be linear. Given that some observational studies suggest adverse child respiratory health outcomes are associated with very high maternal vitamin D level during pregnancy, empiric data are needed before high dose prenatal vitamin D supplementation can be routinely recommended [44].

A limitation of our findings is that healthcare utilization may be too crude, with large health effects required to influence total healthcare utilisation. However, the national framework for health provision in the UK provides a powerful resource of objective information, the merit of which has been validated in a systematic review [45, 46]. When prevalence is based on a combination of diagnosis and prescribing patterns, as we have done, the reliability of this resource further increases [47]. A further limitation of the study is its small size and wide confidence intervals, so that a small effect of prenatal vitamin D on child healthcare utilization in the first 3 years cannot be confidently excluded. However the data were negative when evaluated in different ways, in both adjusted and unadjusted analyses. Overall the data suggest that a clinically meaningful relationship between this regime of vitamin D supplementation in pregnancy in a deficient population, or these levels of vitamin D in cord-blood or at three years of age, and healthcare utilization is unlikely.

Other limitations of this study include the risk of selection bias with follow up rate of only 55% of those randomised. Analyses with imputation for missing data were not undertaken due to the relatively small sample size. However, the characteristics of those included in analysis were similar to those that were excluded. Adjusted analyses were consistent with unadjusted analyses suggesting a low probability that confounding factors have altered the findings of this trial. This was a diverse ethnic group. Variation in healthcare use has been reported between ethnic minorities in the UK, with lower levels of asthma diagnosis and treatment in people from the Indian subcontinent [48, 49] but higher consultation frequency [50, 51]. However, multivariate analysis of our data showed that although maternal ethnicity correlated with 25(OH) vitamin D level there was no relationship between maternal ethnicity and our measures of healthcare utilization in this trial.

In conclusion we have found no evidence that supplementation of a vitamin D deficient, urban mixed race population with either a single oral bolus dose of 200,000 IU cholecalciferol at 27 weeks or 800 IU ergocalciferol daily from 27 weeks until delivery had any effect on child healthcare utilization in the first 3 years of life. Moreover we found no evidence of a relationship between cord blood 25(OH) vitamin D level and healthcare utilization in this population. Although our confidence in the findings is limited by the small sample size, these data suggest that if prenatal vitamin D is used to promote improved child health outcome, then a postnatal component to treatment, and/or or higher doses may be needed.

A recent systematic review of prenatal vitamin D supplementation found heterogeneous study designs and inconsistent findings [52]. Outcomes of a subsequent intervention trial suggest that higher dose vitamin D, and possibly combined mother/infant supplementation may be needed for positive health effects on the child [38]. In that context further data are awaited from two larger randomised controlled trials, VDARRT [53] and ABCVitaminD [54], to establish whether higher dose daily prenatal vitamin D supplementation will impact on child health outcomes.

## Supporting Information

S1 Checklist Consort Checklist. (DOC)

S1 Dataset Data Sheet. (XLSX)

S1 Protocol Trial Protocol. (PDF)

S1 Text Trial Analysis Plan. (PDF)

## References

[1] Scragg R, Camargo CA Jr. Frequency of leisure-time physical activity and serum 25-hydroxyvitamin D levels in the US population: results from the Third National Health and Nutrition Examination Survey. American journal of epidemiology. 2008;168(6):577–86; discussion 87–91. 10.1093/aje/kwn163 18579538 PMC2727193

[2] Wang SS, Hon KL, Kong AP, Pong HN, Wong GW, Leung TF. Vitamin D deficiency is associated with diagnosis and severity of childhood atopic dermatitis. Pediatric allergy and immunology: official publication of the European Society of Pediatric Allergy and Immunology. 2014;25(1):30–5. 10.1111/pai.12167 . 24383670

[3] Wayse V, Yousafzai A, Mogale K, Filteau S. Association of subclinical vitamin D deficiency with severe acute lower respiratory infection in Indian children under 5 y. European journal of clinical nutrition. 2004;58(4):563–7. 10.1038/sj.ejcn.1601845 . 15042122

[4] Camargo CA Jr, Ingham T, Wickens K, Thadhani R, Silvers KM, Epton MJ, et al Cord-blood 25-hydroxyvitamin D levels and risk of respiratory infection, wheezing, and asthma. Pediatrics. 2011;127(1):e180–7. 10.1542/peds.2010-0442 . 21187313

[5] Baiz N, Dargent-Molina P, Wark JD, Souberbielle JC, Annesi-Maesano I, Group EM-CCS. Cord serum 25-hydroxyvitamin D and risk of early childhood transient wheezing and atopic dermatitis. The Journal of allergy and clinical immunology. 2014;133(1):147–53. 10.1016/j.jaci.2013.05.017 . 23810764

[6] Morales E, Romieu I, Guerra S, Ballester F, Rebagliato M, Vioque J, et al Maternal vitamin D status in pregnancy and risk of lower respiratory tract infections, wheezing, and asthma in offspring. Epidemiology. 2012;23(1):64–71. Epub 2011/11/16. 10.1097/EDE.0b013e31823a44d3 . 22082994

[7] Erkkola M, Kaila M, Nwaru BI, Kronberg-Kippila C, Ahonen S, Nevalainen J, et al Maternal vitamin D intake during pregnancy is inversely associated with asthma and allergic rhinitis in 5-year-old children. Clin Exp Allergy. 2009;39(6):875–82. Epub 2009/06/16. CEA3234 [pii] 10.1111/j.1365-2222.2009.03234.x . 19522996

[8] Hansen S, Maslova E, Strom M, Linneberg A, Halldorsson TI, Granstrom C, et al The long-term programming effect of maternal 25-hydroxyvitamin D in pregnancy on allergic airway disease and lung function in offspring after 20 to 25 years of follow-up. The Journal of allergy and clinical immunology. 2015 10.1016/j.jaci.2014.12.1924 . 25649083

[9] Cremers E, Thijs C, Penders J, Jansen E, Mommers M. Maternal and child's vitamin D supplement use and vitamin D level in relation to childhood lung function: the KOALA Birth Cohort Study. Thorax. 2011;66(6):474–80. 10.1136/thx.2010.151985 . 21422038

[10] Pike KC, Inskip HM, Robinson S, Lucas JS, Cooper C, Harvey NC, et al Maternal late-pregnancy serum 25-hydroxyvitamin D in relation to childhood wheeze and atopic outcomes. Thorax. 2012;67(11):950–6. 10.1136/thoraxjnl-2012-201888 22707522 PMC3679514

[11] Wills AK, Shaheen SO, Granell R, Henderson AJ, Fraser WD, Lawlor DA. Maternal 25-hydroxyvitamin D and its association with childhood atopic outcomes and lung function. Clin Exp Allergy. 2013;43(10):1180–8. 10.1111/cea.12172 24074336 PMC3814422

[12] Gupta R, Sheikh A, Strachan DP, Anderson HRi. Burden of allergic disease in the UK: secondary analyses of national databases. Clin Exp Allergy. 2004;34(4):520–6. Epub 2004/04/15. 10.1111/j.1365-2222.2004.1935.x . 15080802

[13] Hollis BW, Johnson D, Hulsey TC, Ebeling M, Wagner CLi. Vitamin D supplementation during pregnancy: double-blind, randomized clinical trial of safety and effectiveness. Journal of bone and mineral research: the official journal of the American Society for Bone and Mineral Research. 2011;26(10):2341–57. Epub 2011/06/28. 10.1002/jbmr.463 21706518 PMC3183324

[14] Goldring ST, Griffiths CJ, Martineau AR, Robinson S, Yu C, Poulton S, et al Prenatal vitamin d supplementation and child respiratory health: a randomised controlled trial. PloS one. 2013;8(6):e66627 10.1371/journal.pone.0066627 23826104 PMC3691177

[15] Berra S, Tebe C, Erhart M, Ravens-Sieberer U, Auquier P, Detmar S, et al Correlates of use of health care services by children and adolescents from 11 European countries. Medical care. 2009;47(2):161–7. Epub 2009/01/27. 10.1097/MLR.0b013e3181844e09 . 19169116

[16] Groholt EK, Stigum H, Nordhagen R, Kohler Li. Health service utilization in the Nordic countries in 1996: Influence of socio-economic factors among children with and without chronic health conditions. European journal of public health. 2003;13(1):30–7. Epub 2003/04/08. . 12678311 10.1093/eurpub/13.1.30

[17] Woodward CA, Boyle MH, Offord DR, Cadman DT, Links PS, Munroe-Blum H, et al Ontario Child Health Study: patterns of ambulatory medical care utilization and their correlates. Pediatrics. 1988;82(3 Pt 2):425–34. Epub 1988/09/01. . 3405678

[18] Starfield B, Hankin J, Steinwachs D, Horn S, Benson P, Katz H, et al Utilization and morbidity: random or tandem? Pediatrics. 1985;75(2):241–7. Epub 1985/02/01. 3969323

[19] Newacheck PWi. Characteristics of children with high and low usage of physician services. Medical care. 1992;30(1):30–42. Epub 1992/01/01. . 1729585 10.1097/00005650-199201000-00003

[20] Ward A, Pratt Ci. Psychosocial influences on the use of health care by children. Australian and New Zealand journal of public health. 1996;20(3):309–16. Epub 1996/06/01. . 8768423 10.1111/j.1467-842x.1996.tb01034.x

[21] Riley AW, Finney JW, Mellits ED, Starfield B, Kidwell S, Quaskey S, et al Determinants of children's health care use: an investigation of psychosocial factors. Medical care. 1993;31(9):767–83. Epub 1993/09/01. . 8366679 10.1097/00005650-199309000-00002

[22] Weissman MM, Warner V, Wickramaratne P, Moreau D, Olfson Mi. Offspring of depressed parents. 10 Years later. Archives of general psychiatry. 1997;54(10):932–40. Epub 1997/10/24. . 9337774 10.1001/archpsyc.1997.01830220054009

[23] Saxena S, Majeed A, Jones Mi. Socioeconomic differences in childhood consultation rates in general practice in England and Wales: prospective cohort study. BMJ. 1999;318(7184):642–6. Epub 1999/03/05. 10066207 10.1136/bmj.318.7184.642 PMC27771

[24] Petrou S, Kupek Ei. Socioeconomic differences in childhood hospital inpatient service utilisation and costs: prospective cohort study. Journal of epidemiology and community health. 2005;59(7):591–7. Epub 2005/06/21. 10.1136/jech.2004.025395 15965144 PMC1757074

[25] Boomla K, Hull S, Robson J. GP funding formula masks major inequalities for practices in deprived areas. BMJ. 2014;349:g7648 10.1136/bmj.g7648 . 25515783

[26] NICE. Antenatal care. Routine care for the healthy pregnant women2008. Available from: http://www.nice.org.uk/nicemedia/live/11947/40145/40145.pdf.

[27] Pearce SH, Cheetham TDi. Diagnosis and management of vitamin D deficiency. BMJ. 2010;340:b5664 Epub 2010/01/13. 10.1136/bmj.b5664 20064851

[28] Yu CK, Sykes L, Sethi M, Teoh TG, Robinson S. Vitamin D deficiency and supplementation during pregnancy. Clinical endocrinology. 2009;70(5):685–90. 10.1111/j.1365-2265.2008.03403.x . 18771564

[29] Iluiu NAO. Provision of out of hours Care 2006. Available from: http://www.nao.org.uk/publications/0506/out-of-hours_care_in_england.aspx.

[30] Curtis L. Unit Costs of Health and Social Care 2009. Mugford ISaM, editor: Personal Social Services Research Unit; 2009.

[31] Government U. Appendix NSRC4 NHS Trust and PCT Combined Reference Cost Schedules 2008/09 In: Health Do, editor. London, United Kingdom 2010.

[32] iluiu LC. Unit Costs of Health and Social Care 2010. McDermid LHaS, editor: Personal Social Services Research Unit; 2010.

[33] Bourn J. The Provision of Out of Hours Care in the UK In: Office NA, editor. United Kingdom: The Stationary Office; 2006.

[34] BNFC. British National Formulary for Children 2011. London: BMJ Group, Pharmaceutical Press, and RCPCH Publications; 2010.

[35] Perneger TV. What's wrong with Bonferroni adjustments. BMJ. 1998;316(7139):1236–8. 9553006 10.1136/bmj.316.7139.1236 PMC1112991

[36] Holick MF, Binkley NC, Bischoff-Ferrari HA, Gordon CM, Hanley DA, Heaney RP, et al Evaluation, treatment, and prevention of vitamin D deficiency: an Endocrine Society clinical practice guideline. The Journal of clinical endocrinology and metabolism. 2011;96(7):1911–30. 10.1210/jc.2011-0385 . 21646368

[37] Ross AC, Manson JE, Abrams SA, Aloia JF, Brannon PM, Clinton SK, et al The 2011 report on dietary reference intakes for calcium and vitamin D from the Institute of Medicine: what clinicians need to know. The Journal of clinical endocrinology and metabolism. 2011;96(1):53–8. 10.1210/jc.2010-2704 21118827 PMC3046611

[38] Grant CC, Kaur S, Waymouth E, Mitchell EA, Scragg R, Ekeroma A, et al Reduced primary care respiratory infection visits following pregnancy and infancy vitamin D supplementation: a randomised controlled trial. Acta paediatrica. 2014 10.1111/apa.12819 . 25283480

[39] BNF. British National Formaulary 62: BMJ Publishing Group Ltd and Royal Pharmaceuticl Society of Great Britain.; 2010 21 Spetember 2011. 1072 p.

[40] Belderbos ME, Houben ML, Wilbrink B, Lentjes E, Bloemen EM, Kimpen JL, et al Cord blood vitamin D deficiency is associated with respiratory syncytial virus bronchiolitis. Pediatrics. 2011;127(6):e1513–20. Epub 2011/05/11. 10.1542/peds.2010-3054 . 21555499

[41] Gynaecology RCoOa. Healthy eating and vitamin supplements in pregnancy. Online: Royal College of Obstectrics and Gynaecology; 2014.

[42] Excellence NIfC. Interventions and advice about diet for women who may become pregnant, or who are pregnant or breastfeeding. Online: NICE; 2015.

[43] Ross AC. The 2011 report on dietary reference intakes for calcium and vitamin D. Public health nutrition. 2011;14(5):938–9. 10.1017/S1368980011000565 . 21492489

[44] Hansen S, Maslova E, Strom M, Linneberg A, Halldorsson TI, Granstrom C, et al The long-term programming effect of maternal 25-hydroxyvitamin D in pregnancy on allergic airway disease and lung function in offspring after 20 to 25 years of follow-up. J Allergy Clin Immunol. 2015;136(1):169–76 e2 10.1016/j.jaci.2014.12.1924 . 25649083

[45] Jordan K, Porcheret M, Croft Pi. Quality of morbidity coding in general practice computerized medical records: a systematic review. Family practice. 2004;21(4):396–412. Epub 2004/07/14. 10.1093/fampra/cmh409 . 15249528

[46] Majeed A, Car J, Sheikh Ai. Accuracy and completeness of electronic patient records in primary care. Family practice. 2008;25(4):213–4. Epub 2008/08/13. 10.1093/fampra/cmn047 . 18694896

[47] Thiru K, Hassey A, Sullivan Fi. Systematic review of scope and quality of electronic patient record data in primary care. BMJ. 2003;326(7398):1070 Epub 2003/05/17. 10.1136/bmj.326.7398.1070 12750210 PMC155692

[48] Duran-Tauleria E, Rona RJ, Chinn S, Burney Pi. Influence of ethnic group on asthma treatment in children in 1990–1: national cross sectional study. BMJ. 1996;313(7050):148–52. Epub 1996/07/20. 8688777 10.1136/bmj.313.7050.148 PMC2351536

[49] Partridge MRi. In what way may race, ethnicity or culture influence asthma outcomes? Thorax. 2000;55(3):175–6. Epub 2000/02/19. 10679533 10.1136/thorax.55.3.175 PMC1745701

[50] Netuveli G, Hurwitz B, Levy M, Fletcher M, Barnes G, Durham SR, et al Ethnic variations in UK asthma frequency, morbidity, and health-service use: a systematic review and meta-analysis. Lancet. 2005;365(9456):312–7. Epub 2005/01/25. 15664226 10.1016/S0140-6736(05)17785-X

[51] Netuveli G, Hurwitz B, Levy M, Fletcher M, Barnes G, Durham SR, et al Ethnic variations in UK asthma frequency, morbidity, and health-service use: a systematic review and meta-analysis. Lancet. 2005;365(9456):312–7. Epub 2005/01/25. 15664226 10.1016/S0140-6736(05)17785-X

[52] Harvey NC, Holroyd C, Ntani G, Javaid K, Cooper P, Moon R, et al Vitamin D supplementation in pregnancy: a systematic review. Health technology assessment. 2014;18(45):1–190. 10.3310/hta18450 25025896 PMC4124722

[53] Litonjua AA, Lange NE, Carey VJ, Brown S, Laranjo N, Harshfield BJ, et al The Vitamin D Antenatal Asthma Reduction Trial (VDAART): rationale, design, and methods of a randomized, controlled trial of vitamin D supplementation in pregnancy for the primary prevention of asthma and allergies in children. Contemporary clinical trials. 2014;38(1):37–50. 10.1016/j.cct.2014.02.006 24614387 PMC4086903

[54] Bisgaard H. Vitamin D Supplementation During Pregnancy for Prevention of Asthma in Childhood (ABCvitaminD) ClinicalTrials.gov: Copenhagen Studies on Asthma in Childhood; 2014 [cited 2014 February 25th]. Available from: https://clinicaltrials.gov/ct2/show/record/NCT00856947.

