# Supplementary material for: Effects of Pre-Natal Vitamin D Supplementation with Partial Correction of Vitamin D Deficiency on Early Life Healthcare Utilisation: A Randomised Controlled Trial
Source: PLoS One. 2015 Dec 23;10(12):e0145303. doi: 10.1371/journal.pone.0145303 (PMC4689556; doi:10.1371/journal.pone.0145303)
Supplement: S1 Protocol — (PDF) [file pone.0145303.s003.pdf]

Imperial College
London

Effects of prenatal vitamin D supplementation on respiratory and allergic phenotypes, and bone density in the first three years of life

Version 3.1

21st October 2010

MAIN SPONSOR:	Imperial College London
FUNDERS:	Asthma UK
STUDY COORDINATION CENTRE:	Imperial College London
REC reference:	10/H0712/13

Protocol authorised by

Name and role	Date	Signature
Robert Boyle, Chief investigator		
Stephen Goldring, Co-investigator		
Professor Warner, Co-investigator		

Imperial College London

This protocol describes the research study and provides information about procedures for entering participants. Every care was taken in its drafting, but corrections or amendments may be necessary. These will be circulated to investigators in the study. Problems relating to this study should be referred, in the first instance, to the Chief Investigator.

This study will adhere to the principles outlined in the NHS Research Governance Framework for Health and Social Care (2nd edition). It will be conducted in compliance with the protocol, the Data Protection Act and other regulatory requirements as appropriate.

Imperial College London

Table of Contents

1. Project management Page 4
2. Study summary Page 5
3. Background Page 6
4. Research questions Page 8
5. Hypothesis Page 8
6. Study objectives Page 8
7. Study design Page 9
8. Primary outcome measure Page 10
9. Secondary outcome measures Page 10
10. Participant entry Page 11
11. Study assessments Page 11
12. Pilot study Page 14
13. Follow up Page 14
14. Adverse events Page 15
15. Statistics and data analysis Page 17
16. Regulatory issues Page 18
    - 16.1 Ethical approval
    - 16.2 Consent
    - 16.3 Confidentiality
    - 16.4 Indemnity
    - 16.5 Sponsor
    - 16.6 Funding
    - 16.7 Audit and inspection
17. Study management Page 19
18. Publication policy Page 20
19. References Page 20
20. Appendices Page 22

1. Project Management

Study Management Group:

Chief Investigator: Dr Robert Boyle

Co-investigators: Dr Stephen Goldring, Dr Nick Harvey, Dr Catherine Hawrylowicz, Dr Adrian Martineau, Dr Stephen Robinson, Dr Seif Shaheen, Professor Cyrus Cooper, Professor Chris Griffiths, Professor Janet Stocks and Professor John Warner

Statistical support: Dr Roger Newson

For clinical queries, general queries, supply of study documentation and collection of data please contact:

Study Coordinator: Dr Stephen Goldring

Address: Wright-Fleming Institute, St Mary's Campus, Imperial College London, London, W2 1PG

Mobile: 07792 923957

Tel: 0207 594 3990

Fax: 0207 594 3984

E-mail: sgoldring@nhs.net

Trial registration: Will register with ISRCTN

Sponsor: Imperial College is the main research sponsor for this study. For further information regarding the sponsorship conditions, please contact the Research Governance Manager at:

Joint Research Office
Sir Alexander Fleming Building
Imperial College
Exhibition Road
London SW7 2AZ
Tel: 020 7594 1188
Fax: 020 7594 1792
http://www3.imperial.ac.uk/clinicalresearchgovernanceoffice

Funder: Asthma UK Grant 09/036

2. Study Summary

**TITLE**	Effects of prenatal vitamin D supplementation on respiratory and allergic phenotypes, and bone density in the first three years of life
**DESIGN**	Prospective follow up of participants of a randomised controlled trial
**AIMS**	To evaluate the effect of prenatal vitamin D supplementation on health outcomes in the first three years of life
**OUTCOME MEASURES**	The primary outcome is the presence or absence of wheezing in the first three years of life. Secondary outcomes include wheezing frequency, atopic sensitisation, allergic disease, bone density, lung function, healthcare utilisation, and markers of immune regulation.
**POPULATION**	Our study population is the offspring of 180 women who attended antenatal clinic at St Mary's hospital in the third trimester of pregnancy and were enrolled in a randomised trial of two methods of vitamin D supplementation(1). (Approved by St Mary's Ethics committee: Ref 06/Q0702/172) In this trial 60 women received 800IU of vitamin D daily from 27 weeks gestation, 60 women received a single injection of 120,000IU of vitamin D at 27 weeks gestation, and 60 women received no vitamin D supplementation. The children were born between 1st June 2007 and 21st November 2007 and we will study this population of children at age three years.
**ELIGIBILITY**	All children whose mothers were enrolled in the prenatal vitamin D study.
**DURATION**	The study involves a single assessment at age 3 years.
**KEYWORDS**	Vitamin D; asthma; child; allergy; wheeze

3. Background

Asthma is the commonest chronic disease of childhood in the United Kingdom. In a recent study the prevalence of asthma in the UK was 20.9% in children aged 6-7 years, and 24.7% in young people aged 13-14 years old (2). Asthma is not curable once it has developed, and in most cases has its origins in early childhood. There is a justified focus on understanding the early life origins of asthma, with a view to developing primary prevention strategies(3).

One possible way to prevent asthma from developing in young children is to give pregnant women vitamin D supplements. There is a plausible rationale for the efficacy of such an approach (4). Four separate cohort studies have found vitamin D intake during pregnancy to be inversely associated with risk of early childhood wheezing in the offspring (5-8). In a North American study, mothers with the highest quartile of vitamin D intake during pregnancy had children with reduced odds for recurrent wheeze (OR 0.39, P<0.001) (5). There was a decrease in these odds of 19% for each 100IU/day increase in estimated daily vitamin D intake during pregnancy, whether such intake was dietary or through supplements. These findings were robust in analyses controlling for a wide variety of potential confounding variables. A separate study in Scotland had remarkably similar findings – vitamin D intake during pregnancy was estimated at 32 weeks gestation. Those mothers with the highest quartile of vitamin D intake had children with decreased risk of persistent wheeze at age 5 compared with those in the lowest quartile (OR 0.33, P=0.01) (6). A third prospective cohort study in Japan of 763 mother infant pairs also described an inverse relationship between maternal vitamin D intake during pregnancy and wheezing at 2 years, with children whose mothers had consumed more than 4.3 micrograms/day of vitamin D having a significantly reduced risk of wheeze and eczema with adjusted odds ratio and 95% confidence intervals of 0.64 [0.43-0.97]) and 0.63 [0.41-0.98] respectively (7). Finally, in a cohort study of 1669 children from Finland, maternal intake of vitamin D from food was negatively related to risk of asthma and allergic rhinitis at 5 years of age, independent of several potential confounders (8).

The Vitamin D receptor is expressed by dendritic cells, T cells and activated B cells, and the immunological effects of vitamin D are diverse, but include an important role in promoting regulatory T cell IL-10 secretion (9). The enzyme for conversion of vitamin D to its active form is expressed on dendritic cells, suggesting a possible role for this vitamin in programming the immune response to environmental antigens (10). Vitamin D also plays an important role in fetal lung development (11-12). If the relationship between vitamin D intake in pregnancy and childhood respiratory outcome is causal then vitamin D may promote respiratory health through either its effects on immune development, its effects on lung development or both.

In view of these associations between vitamin D deficiency and respiratory health, some authors have proposed that vitamin D deficiency may explain the rise in asthma prevalence seen in the past 50 years (13). Certainly vitamin D deficiency is widespread in pregnant women in both the United Kingdom and United States (14) (15). Some of the epidemiology of vitamin D deficiency closely parallels that of asthma epidemiology – for example vitamin D deficiency is common in developed countries, in African-Americans, in recent immigrants to developed countries, in obese individuals and in high latitude countries (4). We are therefore presented with the tantalising prospect of an easily reversible major risk factor for asthma. However the potential role of vitamin D in preventing asthma development is controversial, and some observational studies contradict those cited above(16-18). There is a need for a definitive study to evaluate whether vitamin D supplementation during pregnancy can reduce asthma risk or not.

Prenatal vitamin D also plays a role in postnatal bone mineral accrual (19). In a longitudinal cohort study from Finland (20), risk of hip fracture in adulthood was increased in those born short, and in those who had a low rate of childhood growth, suggesting bone growth is sensitive to adverse intrauterine and early childhood influences (19). Furthermore, reduced concentrations of maternal vitamin D during pregnancy were significantly associated with reduced whole body and lumbar spine bone mineral content in children at 9 years of age in an observational cohort study of children from Southampton (21). In this study, the only two predictors of maternal vitamin D status were estimated sunlight exposure and maternal vitamin D supplementation, which both correlated with whole body bone mineral content at age 9 years in the offspring. Interestingly, the same group have shown that infant feeding practices themselves do not influence bone mass at 4 years of age (22), strengthening the argument that maternal vitamin D status during pregnancy is crucial for programming long term skeletal health. As such, intervention studies to explore the effect of prenatal vitamin D supplementation on bone mineral accrual in children are needed.

4. Research questions

Does prenatal vitamin D supplementation reduce the risk of wheezing illness, atopic disease and healthcare utilisation in offspring at three years of age?
Does prenatal vitamin D supplementation increase bone area and bone density in children at three years of age?

5. Hypothesis

In an ethnically mixed population of women attending for antenatal care in an inner city hospital, vitamin D supplementation during the last trimester of pregnancy promotes fetal lung and immunoregulatory development, causing reduced risk of wheezing illness, atopic disease and healthcare utilisation in offspring at three years of age.

6. Study objectives

The primary objective is to measure the effect of prenatal Vitamin D supplementation on the risk of wheezing in children during the first three years of life.

The secondary objectives of this study are to measure the effect of prenatal vitamin D supplementation at three years of age on the following areas of child health:

1. Lung function
2. Allergic disease (Asthma, eczema, rhinitis, food allergy, atopic sensitisation)
3. Bone density and growth parameters
4. Immune development (Measurement of immune regulation markers)
5. Primary and secondary healthcare utilisation

A further objective is to measure the effect of vitamin D supplementation during pregnancy on maternal vitamin D levels three years after delivery.

7. Study design

A randomised trial of two different methods of vitamin D supplementation during the last trimester of pregnancy has recently been completed by co-investigator Dr Robinson (1). This trial evaluated the relative effectiveness of two different methods of vitamin D supplementation during pregnancy on increasing vitamin D levels at delivery in both the mother and the newborn. 180 women were recruited from an ethnically diverse population attending antenatal clinics at St Mary's Hospital, Imperial College Healthcare NHS Trust. After informed consent, women at 27 weeks gestation were randomly allocated into one of three arms: daily supplementation with 800IU of vitamin D (n=60), a single injection of 120,000IU vitamin D (n=60), and no intervention (n=60). The children were born between 1st June 2007 and 21st November 2007. The results of this study have been published and show statistically significant increases in both maternal and neonatal 25-hydroxyvitamin D levels in the intervention groups (1). For the recruited women and their newborns, this trial ended at delivery.

This follow up study aims to assess the effect of pre-natal vitamin D supplementation during pregnancy on child health by inviting the mothers who participated in the above trial to bring their children to Imperial College Paediatric Research Unit at St. Mary's Hospital for a single clinical assessment. We will carry out the assessments at three years of age.

Each child's assessment will include:
1. Clinical history using a structured questionnaire
2. Clinical examination including growth assessment and assessment of skin darkness
3. Lung function tests
4. Skin tests
5. Nasal secretion sampling for inflammatory markers
6. Blood test for immune function markers and RNA/ DNA extraction for future genetic and epigenetic work
7. DXA (Dual-energy X-ray Absorptiometry) scan to measure bone size and density
8. Buccal (mouth) swab for DNA in those who do not consent to a blood test

In addition, each mother will be invited to have the following investigations
1. Assessment of skin darkness
2. Blood test to assess their vitamin D status
3. Blood test for DNA extraction with a view to future testing to evaluate whether certain genetic polymorphisms modify any effects of prenatal vitamin D supplementation on outcomes in mother or child.

4. Buccal (mouth) swab for DNA in those that do not consent to a blood test

9. Primary outcome measure

1. Percentage (%) of children with any wheezing episode in the first 3 years of life.

10. Secondary outcome measures

1. % of children with any wheezing episode in the preceding 12 months
2. % of children using inhaled bronchodilators in the last 12 months
3. Total number of all wheezing episodes since birth
4. **%** of children with doctor diagnosed asthma
5. % of children with doctor diagnosed eczema either prior to or at the time of the assessment
6. % of children with doctor diagnosed rhinitis either prior to or at the time of the assessment
7. % of children with doctor diagnosed food allergy
8. Total number of upper and lower respiratory tract infections since birth
9. % of children with positive skin prick test responses to one or more of a panel of common allergens
10. Pulmonary airflow resistance at a range of frequencies using impulse oscillometry
11. Change in pulmonary airflow resistance after salbutamol inhalation, using impulse oscillometry
12. Rebreathing exhaled nitric oxide level (in parts per billion) at study assessment
13. Whole body minus head bone area, bone mineral content, areal bone mineral density and estimated volumetric bone mineral density using DXA instrument
14. Sitting and standing height, weight and head circumference
15. 25-hydroxyvitamin D and PTH levels
16. Fasting blood for serum CTX/P1NP
17. Nasal secretions for inflammatory (e.g. IL4, IL5, IL13, eotaxin, RANTES TNF-alpha) and anti-inflammatory (e.g. IL-10, TGF-beta) mediators
18. Total and specific IgE
19. Eosinophil count
20. Total IgG, A, M and IgG subclass levels
21. Tetanus and Hib IgG antibody levels
22. CD4 T cell proliferative response to recombinant DerP1
23. Soluble ICAM-1 and Serum Soluble IL-2R
24. Dendritic cell numbers and phenotype, and $FoxP3^{hi}$ CD4 T cell numbers by flow cytometry
25. Infant RNA/DNA (Store)
26. Maternal vitamin D and PTH levels and maternal DNA

27. Number of unscheduled healthcare visits in the first 3 years of life

11. Participant entry

**Pre-registration evaluation**	No tests are required before a participant can enter the study
**Inclusion criteria**	All children born of mothers enrolled in the previous trial (1) are eligible to be enrolled in the study
**Exclusion criteria**	Severe congenital or developmental abnormalities likely to significantly affect respiratory health or lung function eg congenital thoracic dystrophy
**Withdrawal criteria**	None – the study consists of a single clinical assessment. Parents and children who wish to end the assessment may do so at any time.

12. Study assessment

The study consists of a single assessment of the child. Parents and their children who agree to take part in the study will be invited to attend the Paediatric Research Unit at St Mary's hospital at age three years. After informed consent is taken from the parent/guardian a member of the research team will perform the assessment as follows, which is expected to take less than two hours.

11.1 Structured interview

The research clinician will take a medical history from the child's parent/guardian using a structured questionnaire to obtain information on primary and secondary clinical outcomes. This is expected to take approximately 20 minutes. The primary outcome measure is a history of wheezing at any time during the first 3 years of life. We will support this by demonstration of a child wheezing in a short video.

In addition, parent(s) will also answer a validated questionnaire about their own respiratory and allergic health, their risk factors for vitamin D deficiency, and environmental risk factors for wheezing in their child.

11.2 Clinical examination

Children will have their height, weight and head circumference measured and have a clinical examination, including examination of the skin for eczema using the UK Working Party definition for eczema diagnosis. Where eczema is present its severity will be documented using a validated scoring system. The chest and nose will be examined for signs of lung disease and rhinitis. Skin darkness will be measured using a handheld

colorimeter probe placed gently against the skin. This probe measures melanin levels which are known to be a major determinant of vitamin D absorption and status (23).

11.3 Lung function tests

Children will have their lung function measured using impulse oscillometry before and 15 minutes after administration of salbutamol via a spacer. This technique has been shown in several studies to be reliable and valid in children as young as 2 years, (24-26), and is able to detect significant bronchodilator responses which are predictive of asthma (27).

To perform the test the child will be seated comfortably with their head in a neutral position. Using an adjustable arm, the mouthpiece is brought level with the child's mouth. A nose clip is placed on the subject and the child will be encouraged to breathe normally through the mouthpiece. To minimise interference from vibrations in the upper airways the child's cheeks will be firmly supported by their parent/ guardian. Data acquisition should cover several normal breathing cycles like this, usually between 8 and 16 seconds. The mean value of three to five technically acceptable measurements is reported, and usually takes about one minute to acquire (28). 15 minutes after administration of salbutamol delivered via spacer device fitted with a face mask, measurements will be repeated.

11.4 Measurement of exhaled nitric oxide (eNO)

Children will have their exhaled nitric oxide level measured using an offline tidal volume technique, which has been shown in several studies to be a reliable and valid method in children as young as two years (26, 29).

12.5 Allergy skin tests for atopic sensitisation

Children will undergo allergy skin testing to a panel of common allergens including house dust mite, cat dander, grass pollen, alternaria and cladosporum, silver birch pollen, cow's milk, egg and peanut. Atopy is defined as a positive response to at least one allergen, and a positive response is defined as a wheal diameter ≥3 mm in the presence of appropriate responses to the negative and positive control.

11.6 Collection of blood samples

If parents/ guardians consent, a blood sample will also be taken from each child. To reduce discomfort a local anaesthetic cream will be applied. A total of 10mls of blood will be taken – 1.5ml EDTA for future DNA extraction, 3ml Tempus tube for future RNA extraction, 2mls for serum separation, and 3.5mls for flow cytometry and cell culture on fresh mononuclear cells.

11.7 Collection of nasal secretions

If parents/ guardians consent, a sample of nasal secretions will also be taken using a filter paper technique established in our department, for evaluation of inflammatory cytokines IL4, IL5, IL13, eotaxin and RANTES (30). For this procedure a small piece of synthetic absorptive matrix (SAM) will be placed in the nostrils of participating children and left in position for 2 minutes to absorb nasal epithelial lining fluid. SAM are subsequently spin filtered and eluate is analysed by multiplex cytokine assay for inflammatory mediators (Luminex Corporation, Austin, TX).

11.8 DXA scan for bone density

A DXA scan uses low energy x-rays from two different sources to measure the density of the bone being tested. To perform this test, children and their parents will be taken to the radiology department of St Mary's hospital accompanied by the research clinician. Children will all be asked to wear similar light clothing, to reduce any artefactual increase in bone density attributable to their clothes. During the test, the child lies still on their back on a couch while an x-ray detector arm (the 'scanner') sweeps over the area to be tested. The whole scan takes 3-4 minutes. During this time age appropriate distraction (for example a video or book) will be provided to maintain cooperation of the child. 'Whole body minus head' bone area, bone mineral content, areal bone mineral density and estimated volumetric bone mineral density will be assessed – in previous studies by co-investigators Nick Harvey and Cyrus Cooper, 95% of children in this age group were able to cooperate sufficiently well to yield a good quality bone mineral density measurement. The radiation exposure related to this procedure for the child is equivalent to 4 days of normal daily background radiation exposure.

11.9 Review of healthcare records

Permission will be sought from each participant to review their child's primary healthcare records held by their General Practitioner, in order to assess healthcare utilisation in relation to respiratory illness and the total number of unplanned healthcare attendances either to a General Practitioner or an Emergency Department during the first 3 years of life.

11.10 Assessment of mothers

If the mother consents, we will assess their skin darkness using the colorimeter probe and collect a blood sample for vitamin D status and DNA extraction.

11.11 Home or telephone assessments

If families are unable to visit for these assessments, they will be offered a home visit by the research clinician to obtain clinical outcomes. If families decline, questionnaires will be administered over the telephone in order to maximise data acquisition for the outcome measures.

11.12 Permission will also be sought to take a buccal swab from both mother and child for DNA analysis. This test involves using a special cotton bud to gently stroke the inside of the mouth to collect cells from the mouth which can then be used to extract DNA. This is especially useful for any mothers/ children who would rather not have a blood test. The test does not hurt.

13. Pilot study

Prior to enrolment of the first patient we will run a pilot study in 60 children with and without a history of pre-school wheezing recruited from the community and from paediatric outpatients at St Mary's hospital. The aim of this pilot study will be to establish local reference values for respiratory resistance and bronchodilator response to salbutamol measured using impulse oscillometry, as advised by the American Thoracic society/ European Task Force statement on pulmonary function testing in pre-school children (28). Given that the majority of published reference ranges for impulse oscillometry in young children have obtained measurements in white children (31) , with small numbers of three year olds, it is important that a representative sample in terms of age and ethnicity is obtained. To better interpret these data we will also perform a short questionnaire based interview regarding respiratory symptoms, measure height and weight, skin darkness using the colorimeter probe, inflammatory cytokines in nasal secretions using the filter paper technique and atopic sensitisation by performing an allergy skin test to common allergens.

14. Follow up

Although the study consists of a single assessment at 3 years of age, will ask mothers if they would be willing to be contacted in the future. If we find evidence to suggest that vitamin D supplementation is associated with a reduction in wheezing in the offspring, it would be of value to follow-up the children three years later in order to whether the effects of prenatal vitamin D persist beyond the first 3 years of life.

To minimise bias, all investigators and research staff involved in data collection or analysis for this follow up study will be blinded to the prenatal treatment allocation of the study participants, and participants will be asked not to mention their treatment allocation to the research team at their visit. Staff will also be blind to the results of the maternal and cord blood vitamin D levels until primary analyses have been completed, and any

episodes of inadvertent unblinding prior to data analysis due to maternal comments will be documented and reported in publications.

14. Adverse events

14.1 Definitions

Adverse Event (AE): any untoward medical occurrence in a patient or clinical study subject which occurs during the assessment visit.

Serious Adverse Event (SAE): any untoward and unexpected medical occurrence or effect that:

- Results in death
- Is life-threatening – *refers to an event in which the subject was at risk of death at the time of the event; it does not refer to an event which hypothetically might have caused death if it were more severe*
- Requires hospitalisation, or prolongation of existing inpatients' hospitalisation
- Results in persistent or significant disability or incapacity
- Is a congenital anomaly or birth defect

Medical judgement should be exercised in deciding whether an AE is serious in other situations. Important AEs that are not immediately life-threatening or do not result in death or hospitalisation but may jeopardise the subject or may require intervention to prevent one of the other outcomes listed in the definition above, should also be considered serious.

All adverse events will be reported, and the reporting procedures below will be followed. Any questions concerning adverse event reporting should be directed to the Chief Investigator in the first instance.

14.2 Non serious AEs

All such events, whether expected or not, should be recorded.

14.3 Serious AEs

An SAE form should be completed and faxed to the Chief Investigator within 24 hours.

All SAEs will be reported to the St Mary's Hospital Research and Ethics Committee where in the opinion of the Chief Investigator, the event was:

- 'related', i.e. resulted from the administration of any of the research procedures; and
- 'unexpected', i.e. an event that is not listed in the protocol as an expected occurrence

Reports of related and unexpected SAEs will be submitted within 15 days of the Chief Investigator becoming aware of the event, using the NRES form for non-IMP studies.

Local investigators will also report any SAEs to the Imperial College Joint Research Office and local Research Ethics Committee.

Contact details for reporting SAEs

Fax: 0207 594 3984, attention Robert Boyle

Please send SAE forms to: Robert Boyle

Tel: 0207 594 3990 (Mon to Fri 09.00 – 17.00)

15. Statistics and data analysis

An estimated 80% of study participants will be successfully evaluated in this study for the primary outcome measure, with an estimated wheeze prevalence of 34% at 3 years in the children of placebo-treated mothers (32). We will therefore have 80% power to detect a reduction in risk of wheeze to 13% (risk ratio of 0.38) in the children of vitamin D treated mothers at the 5% level of significance This level of risk reduction would be consistent with that seen in the observational studies (5, 32). Primary analyses will evaluate the effects of either form of vitamin D supplementation during pregnancy on outcome measures using available case analysis without imputation. The strength of any positive findings will be explored using imputation for missing data if greater than 10% of subjects are lost to follow up for the primary outcome measure. Secondary analyses will evaluate the relationship between cord blood 25-hydroxyvitamin D levels and outcome measures of interest. Analyses will use logistic regression to evaluate the effects of the intervention or cord blood levels on binary outcome measures, and linear regression to analyse continuous outcome measures. If there is chance imbalance between the arms with respect to potential confounding factors we will control for these in the regression models.

Data and all appropriate documentation will be stored for a minimum of 10 years after the completion of the study.

16. Regulatory issues

16.1 Ethics approval

The Chief Investigator has obtained approval from the St Mary's Hospital Research Ethics Committee (REC reference 10/H0712/13). The study has also been submitted for Site Specific Assessment (SSA) at Imperial College Healthcare NHS Trust. The Chief Investigator will require a copy of the SSA approval letter before accepting participants into the study. The study will be conducted in accordance with the recommendations for physicians involved in research on human subjects adopted by the 18th World Medical Assembly, Helsinki 1964 and later revisions.

16.2 Consent

Consent to enter the study will be sought from each participant only after a full explanation has been given, an information leaflet offered and time allowed for consideration. Consent will be obtained from a parent or guardian of each child enrolled in the study. This will be signed consent for children attending the paediatric research unit for a study assessment. Where parents or guardians agree to answer the questionnaire over the telephone, but not to attend for a health assessment, verbal consent to take part in the study, witnessed by a second researcher will be obtained and recorded on the consent form. The right of the parent or participant to refuse to participate without giving reasons will be respected. After the participant has entered the study the clinician remains free to give alternative treatment to that specified in the protocol at any stage if he/she feels it is in the participant's best interest, but the reasons for doing so should be recorded. In these cases the participants remain within the study for the purposes of follow-up and data analysis. All parents and participants are free to withdraw at any time from the protocol treatment without giving reasons and without prejudicing further treatment.

16.3 Confidentiality

The Chief Investigator will preserve the confidentiality of participants taking part in the study and is registered under the Data Protection Act.

16.4 Indemnity

Imperial College London holds negligent harm and non-negligent harm insurance policies which apply to this study.

16.5 Sponsor

Imperial College London will act as the main sponsor for this study. Delegated responsibilities will be assigned to the NHS trust taking part in this study.

16.6 Funding

Asthma UK is funding this study (grant 09/036).

Participant travel and parking expenses will be reimbursed for return travel by public transport or personal vehicle to the Paediatric Research Unit, St. Mary's Hospital but taxi travel will not be reimbursed.

16.7 Audit and inspection

The study may be subject to inspection and audit by Imperial College London under their remit as sponsor and other regulatory bodies to ensure adherence to GCP and the NHS Research Governance Framework for Health and Social Care (2nd edition).

17. Study management

The day-to-day management of the study will be co-ordinated through Dr Stephen Goldring, Clinical Research Fellow in the Department of Paediatrics, Imperial College London St. Mary's campus. No formal Data or Safety Management Board has been established for this non-interventional study. However, the study team will meet on a regular basis to review its progress.

18. Publication policy

Results of this study will be published in the scientific peer-reviewed literature, and presented at national and international congresses relevant to asthma and allergic disease. Results will also be disseminated to asthma patients via the funder Asthma UK.

19. References

1. Yu CK, Sykes L, Sethi M, Teoh TG, Robinson S. Vitamin D deficiency and supplementation during pregnancy. Clin Endocrinol (Oxf). 2009 May;70(5):685-90.
2. Asher MI, Montefort S, Bjorksten B, Lai CK, Strachan DP, Weiland SK, et al. Worldwide time trends in the prevalence of symptoms of asthma, allergic rhinoconjunctivitis, and eczema in childhood: ISAAC Phases One and Three repeat multicountry cross-sectional surveys. Lancet. 2006 Aug 26;368(9537):733-43.
3. AsthmaUK. Basic asthma research II: Asthma UK;2006. . 2006.
4. Litonjua AA, Weiss ST. Is vitamin D deficiency to blame for the asthma epidemic? J Allergy Clin Immunol. 2007 Nov;120(5):1031-5.
5. Camargo CA, Jr., Rifas-Shiman SL, Litonjua AA, Rich-Edwards JW, Weiss ST, Gold DR, et al. Maternal intake of vitamin D during pregnancy and risk of recurrent wheeze in children at 3 y of age. clinical study Am J Clin Nutr. 2007 Mar;85(3):788-95.
6. Devereux G, Litonjua AA, Turner SW, Craig LC, McNeill G, Martindale S, et al. Maternal vitamin D intake during pregnancy and early childhood wheezing. Am J Clin Nutr. 2007 Mar;85(3):853-9.
7. Miyake Y, Sasaki S, Tanaka K, Hirota Y. Dairy food, calcium, and vitamin D intake in pregnancy and wheeze and eczema in infants. Eur Respir J. 2009 Oct 19.
8. Erkkola M, Kaila M, Nwaru BI, Kronberg-Kippila C, Ahonen S, Nevalainen J, et al. Maternal vitamin D intake during pregnancy is inversely associated with asthma and allergic rhinitis in 5-year-old children. Clin Exp Allergy. 2009 Jun;39(6):875-82.
9. Xystrakis E, Kusumakar S, Boswell S, Peek E, Urry Z, Richards DF, et al. Reversing the defective induction of IL-10-secreting regulatory T cells in glucocorticoid-resistant asthma patients. J Clin Invest. 2006 Jan;116(1):146-55.
10. Hewison M, Burke F, Evans KN, Lammas DA, Sansom DM, Liu P, et al. Extra-renal 25-hydroxyvitamin D3-1alpha-hydroxylase in human health and disease. J Steroid Biochem Mol Biol. 2007 Mar;103(3-5):316-21.
11. Nguyen TM, Guillozo H, Marin L, Tordet C, Koite S, Garabedian M. Evidence for a vitamin D paracrine system regulating maturation of developing rat lung epithelium. Am J Physiol. 1996 Sep;271(3 Pt 1):L392-9.
12. Nguyen M, Trubert CL, Rizk-Rabin M, Rehan VK, Besancon F, Cayre YE, et al. 1,25-Dihydroxyvitamin D3 and fetal lung maturation: immunogold detection of VDR expression in pneumocytes type II cells and effect on fructose 1,6 bisphosphatase. J Steroid Biochem Mol Biol. 2004 May;89-90(1-5):93-7.
13. Weiss ST, Litonjua AA. Maternal diet vs lack of exposure to sunlight as the cause of the epidemic of asthma, allergies and other autoimmune diseases. Thorax. 2007 Sep;62(9):746-8.
14. Hypponen E, Power C. Hypovitaminosis D in British adults at age 45 y: nationwide cohort study of dietary and lifestyle predictors. Am J Clin Nutr. 2007 Mar;85(3):860-8.
15. Bodnar LM, Simhan HN, Powers RW, Frank MP, Cooperstein E, Roberts JM. High prevalence of vitamin D insufficiency in black and white pregnant women residing in the northern United States and their neonates. J Nutr. 2007 Feb;137(2):447-52.
16. Gale CR, Robinson SM, Harvey NC, Javaid MK, Jiang B, Martyn CN, et al. Maternal vitamin D status during pregnancy and child outcomes. Eur J Clin Nutr. 2008 Jan;62(1):68-77.
17. Shaheen SO. Vitamin D deficiency and the asthma epidemic. Thorax. 2008 Mar;63(3):293; author reply
18. Hypponen E, Sovio U, Wjst M, Patel S, Pekkanen J, Hartikainen AL, et al. Infant vitamin d supplementation and allergic conditions in adulthood: northern Finland birth cohort 1966. Ann N Y Acad Sci. 2004 Dec;1037:84-95.
19. Cooper C, Javaid K, Westlake S, Harvey N, Dennison E. Developmental origins of osteoporotic fracture: the role of maternal vitamin D insufficiency. The Journal of Nutrition. 2005;135(11):2728S-34S.

20. Cooper C, Eriksson JG, Forsen T, Osmond C, Tuomilehto J, Barker DJ. Maternal height, childhood growth and risk of hip fracture in later life: a longitudinal study. Osteoporos Int. 2001;12(8):623-9.
21. Javaid MK, Crozier SR, Harvey NC, Gale CR, Dennison EM, Boucher BJ, et al. Maternal vitamin D status during pregnancy and childhood bone mass at age 9 years: a longitudinal study. The Lancet. 2006;367(9504):36-43.
22. Harvey NC, Robinson SM, Crozier SR, Marriott LD, Gale CR, Cole ZA, et al. Breast-feeding and adherence to infant feeding guidelines do not influence bone mass at age 4 years. Br J Nutr. 2009 Sep;102(6):915-20.
23. Holick MF. Vitamin D deficiency. N Engl J Med. 2007 Jul 19;357(3):266-81.
24. Hall GL, Sly PD, Fukushima T, Kusel MM, Franklin PJ, Horak F, Jr., et al. Respiratory function in healthy young children using forced oscillations. Thorax. 2007 Jun;62(6):521-6.
25. Oostveen E, MacLeod D, Lorino H, Farre R, Hantos Z, Desager K, et al. The forced oscillation technique in clinical practice: methodology, recommendations and future developments. Eur Respir J. 2003 Dec;22(6):1026-41.
26. Daniel PF, Klug B, Valerius NH. Measurement of exhaled nitric oxide in young children during tidal breathing through a facemask. Pediatr Allergy Immunol. 2005 May;16(3):248-53.
27. Marotta A, Klinnert MD, Price MR, Larsen GL, Liu AH. Impulse oscillometry provides an effective measure of lung dysfunction in 4-year-old children at risk for persistent asthma. J Allergy Clin Immunol. 2003 Aug;112(2):317-22.
28. Beydon N, Davis SD, Lombardi E, Allen JL, Arets HG, Aurora P, et al. An official American Thoracic Society/European Respiratory Society statement: pulmonary function testing in preschool children. Am J Respir Crit Care Med. 2007 Jun 15;175(12):1304-45.
29. Pijnenburg MW, De Jongste JC. Exhaled nitric oxide in childhood asthma: a review. Clin Exp Allergy. 2008 Feb;38(2):246-59.
30. Erin EM, Zacharasiewicz AS, Nicholson GC, Tan AJ, Higgins LA, Williams TJ, et al. Topical corticosteroid inhibits interleukin-4, -5 and -13 in nasal secretions following allergen challenge. Clin Exp Allergy. 2005 Dec;35(12):1608-14.
31. Frei J, Jutla J, Kramer G, Hatzakis G, Ducharme F, Davis GM. Impulse oscillometry: reference values in children 100 to 150 cm in height and 3 to 10 years of age. Chest. 2005;128(3):1266-73.
32. Martinez FD, Wright AL, Taussig LM, Holberg CJ, Halonen M, Morgan WJ. Asthma and wheezing in the first six years of life. The Group Health Medical Associates. N Engl J Med. 1995 Jan 19;332(3):133-8.

20. Appendices

Appendix 1	Invitation letter to participants (Main study)
Appendix 2	Participant information sheet (Main study)
Appendix 3	Consent form for mother (Main study)
Appendix 4	Consent form for parents/guardian of child (Main study)
Appendix 5	GP letter (Main study)

Pilot study

Appendix 6	Participant information sheet (Pilot study)
Appendix 7	Consent form for parents/ guardian of child (Pilot study)
Appendix 8	GP letter (Pilot study)
