# Supplementary material for: Effects of Pre-Natal Vitamin D Supplementation with Partial Correction of Vitamin D Deficiency on Early Life Healthcare Utilisation: A Randomised Controlled Trial
Source: PLoS One. 2015 Dec 23;10(12):e0145303. doi: 10.1371/journal.pone.0145303 (PMC4689556; doi:10.1371/journal.pone.0145303)
Supplement: S1 Text — (PDF) [file pone.0145303.s004.pdf]

Prenatal vitamin D study
Statistical Analysis Plan

Approved Version 1.0
Date 6/06/2011

Approved by TMG:

Signature (Chair): ____________________________ Date: ___/___/______

Approved by DMC:

Signature (Chair): ____________________________ Date: ___/___/______

Approved by TSC:

Signature (Chair): ____________________________ Date: ___/___/______

Trial Statisticians: Richard Hooper

This document was created based on the Mental Health & Neuroscience Clinical Trials Unit (MH&N CTU) Analysis Strategy template (version 1.5; 13/02/2008) based on an earlier version developed by Rebecca Walwyn and Sally Lee.

1 INTRODUCTION

1.1 Purpose of statistical analysis plan

The purpose of this document is to provide details of the statistical analyses and presentation of results to be reported within the principal paper(s) of the prenatal vitamin D supplementation trial. Subsequent papers of a more exploratory nature (including those involving baseline data only) will not be bound by this strategy but will be expected to follow the broad principles laid down in it. Any exploratory, post-hoc or unplanned analyses will be clearly identified in the respective study analysis report.

The structure and content of this document provides sufficient detail to meet the requirements identified by the International Conference on Harmonisation (ICH) and the PCTU SOP (PCTU/07).

The following were reviewed in preparation for writing this document:
Trial protocol registered at ISRCTN 68645785
ICH E9 Guidance on statistical principals for clinical trials
ICH E3 Structure and content of clinical study reports
CONSORT guidelines for the reporting of randomised trials, including trials of non-pharmacological treatments

1.2 Members of the writing committee

Section should detail who has been involved in developing the analysis strategy, responsible for writing it and authorship of papers if required

Dr Stephen Goldring, Dr Robert Boyle, Professor Seif Shaheen, Professor Chris Griffiths and Professor John Warner were primarily responsible for (i) writing the Statistical Analysis Strategy with Dr Richard Hooper responsible for overseeing the analyses.

This document has been developed prior to unblinding of trial data and will not be implemented prior to final approval.

1.3 Summary

Hypothesis

Vitamin D supplementation during the last trimester of pregnancy reduces risk of wheezing illness, eczema, atopy and healthcare utilisation in offspring during the first three years of life.

Study design

This is a prospective randomised controlled trial of vitamin D supplementation during pregnancy. In this study (Yu, Sykes et al. 2009), 180 women who attended antenatal clinic at St Mary's hospital in the third trimester of pregnancy were randomised at 27 weeks to receive either 800IU of vitamin D daily until delivery (60 women), a single oral

dose of 200,000IU of vitamin D (60 women) or no vitamin D supplementation (60 women). This statistical analysis plan pertains to analysis of the offspring of randomized women, who were born between 1st June 2007 and 21st November 2007, to evaluate the effect of prenatal vitamin D supplementation on the prevalence of ≥1 episode of wheezing during the first three years of life. Secondary outcomes include wheezing frequency, asthma predictive index, respiratory infection, atopic sensitisation, eczema, lung function parameters, healthcare utilisation, and laboratory markers of immune regulation.

1.4 Changes from planned analysis in the protocol

The study protocol included plans for analysis of child bone densitometry, serological markers of bone turnover, maternal vitamin D and PTH levels, child total and specific immunoglobulin levels, child nasal inflammometry and CD4 T cell proliferative response to antigen, soluble ICAM and IL-2R. All these analyses were subsequently excluded from the study, due to financial or logistical limitations, or in the case of nasal inflammometry due to poor acceptability of this procedure by 3 year old children during the pilot phase of the project.

2 STUDY OBJECTIVES AND ENDPOINTS

2.1 Study objectives

2.1.1 Primary objectives

Does prenatal vitamin D supplementation reduce the risk of wheezing in the first 3 years of life?

2.1.2 Secondary objectives

Does prenatal vitamin D supplementation reduce the risk of atopic and non-atopic wheezing, change physiological markers of lung function, reduce the frequency of eczema, atopy and other allergic outcomes, frequency of respiratory infections, or overall healthcare utilization in children in the first 3 years of life?

2.1.3 Exploratory objectives

Are the effects of the intervention on wheezing, atopy or eczema modified by parental atopy or baseline maternal vitamin D status?
Is cord blood vitamin D status related to risk of wheezing in the first 3 years of life?

2.1.4 Primary outcome

1. **Number of children with any episode of wheezing (Ever wheezing)**

2.1.5 Secondary outcomes

1. **Wheezing related**

a. Recurrent wheezing (ie more than 1 episode of parent reported wheezing)
b. Wheezing episodes in the past 12 months
c. Ever wheezing in children with and without a positive Asthma predictive index
d. Any prescription of inhaled bronchodilators
e. Physiological markers of lung function measured using impulse oscillometry pre and post bronchodilator (Resonant frequency (fr), area under reactance curve and resistance at 10 and 20 Hz
f. Exhaled nitric oxide (ppb)
g. Blinded consultant paediatrician's assessment of recurrent wheeze using primary care electronic health record

2. **Eczema and atopy related**

a. Eczema ever
b. Eczema in the past 12 months
c. Atopy
d. Total IgE
e. Allergic rhinitis
f. Food allergy (doctor diagnosed)
g. Blinded consultant paediatrician's assessment of eczema using primary care electronic health record

h. Blinded consultant paediatrician’s assessment of food allergy using primary care electronic health record

3. Infection related

a. Frequency of upper respiratory tract infections
b. Frequency of lower respiratory tract infections

4. Healthcare cost related

a. Total healthcare costs to the NHS, estimated from GP electronic health record
b. Total healthcare attendance costs related to respiratory illness
c. Total healthcare attendance costs related to allergic illness
d. Total healthcare costs in the first year of life

5. Exploratory analysis

a. We will explore if the effects of the intervention are modified by parental atopy and baseline maternal vit D
b. We will explore the relationship of cord 25(OH)D to primary and secondary outcomes.

2.1.6 Safety outcomes

1. Growth – height, weight and BMI z scores at 3 years
2. Congenital malformations
3. Gestation
4. Birthweight
5. Haematological parameters at age 3 years (Haemaglobin, MCV, white cell count, neutrophil count, lymphocyte count, platelet count)
6. Neonatal unit admission
7. Death – stillbirth/neonatal death/postneonatal death, and causes
8. Chronic non-atopic health condition in child (Q8)

3 STUDY METHODS

3.1 Overall study design and plan

Study design

Prospective follow up of the **offspring** of a randomised controlled trial of vitamin D supplementation during pregnancy (Yu, Sykes et al. 2009).

Study population

The offspring of 180 women who attended antenatal clinic at St Mary's hospital and participated in the above study. This population were born between 1st June 2007 and 21st November 2007. Pregnant women from the following ethnic populations were included; 45 Indian Asians, 45 Middle Eastern, 45 Black and 45 Caucasian women.

Inclusion criteria

All mothers and their offspring were contacted and invited to participate,

Exclusion criteria

None

Intervention

Women were randomly allocated at 27 weeks gestation as follows:
A daily dose of 800IU vitamin D (ergocalciferol) until delivery (60 women)
A single oral dose of 200,000IU of vitamin D (Calciferol) at 27 weeks (60 women)
No vitamin D supplementation (60 women).

Blinding of original intervention

Study personnel and participants were not blinded to treatment assignment. The person seeing the pregnant women allocated the next available number on entry to the trial, and each woman collected her tablets directly from the hospital pharmacy department or her local pharmacy. All women were given instructions to avoid other multivitamin supplements that would lead to higher vitamin D intake.

Blinding of follow up study

All researchers participating in the follow up study were **blinded** as to the treatment allocation of the mothers.

3.2 Selection of study population

All offspring of the original study were invited to participate. The trial recruited pregnant women from St. Mary's hospital antenatal clinic. Inclusion criteria were pregnant women from the following ethnic populations; 45 Indian Asians, 45 Middle Eastern, 45 Black and 45 Caucasian women. Women who did not speak English were only included if a health advocate was able to interpret and a leaflet was provided in their language. Women with pre-existing sarcoidosis, osteomalacia, renal dysfunction and tuberculosis were excluded from the study.

3.3 Method of treatment assignment and randomisation

Subjects were randomized within each ethnic group. Computer generated random number lists were drawn up by an independent researcher, with randomization in blocks of 15. Randomization was stratified by ethnic group, and participants were allocated to daily vitamin D, bolus vitamin D and no treatment at a 1:1:1 ratio. Treatment was allocated by a third party not involved in enrolment or outcome assessment (hospital pharmacy) according to the randomization list.

3.4 Treatment masking (Blinding)

The original intervention during pregnancy was unblinded.

All researchers involved in the follow up study were blinded to the original allocation group.

Mothers were told by letter and verbally not to reveal their treatment allocation to study staff.

3.5 Sample size determination

It was estimated that 80% of study participants would be successfully evaluated in this study for the primary outcome measure, with an estimated wheeze prevalence of 34% at 3 years in the children of placebo-treated mothers (Martinez, Wright et al. 1995). We would therefore have 80% power to detect a reduction in risk of wheeze to 13% (risk ratio of 0.38) in the children of vitamin D treated mothers at the 5% level of significance This level of risk reduction would be consistent with that seen in the observational studies (Martinez, Wright et al. 1995; Camargo, Rifas-Shiman et al. 2007).

DATA COLLECTION

3.6 Baseline

Pre-intervention

Maternal ethnicity
Maternal 25-hydroxyvitamin D, Calcium, PTH

At delivery

Maternal 25-hydroxyvitamin D
Cord blood 25-hydroxyvitamin D

3.7 Follow up

A copy of the questionnaire and record of physical assessment is attached as an appendix to this document.

3.7.1 Respiratory tract infections

Lower respiratory tract infection was defined as a positive response to the question "Since your child was born have you ever been told by a health professional that your child has bronchiolitis, bronchitis, croup, pneumonia or a chest infection?" Subsequent questions inquired about the management of any such episodes including attendance at A+E, admission overnight to hospital, and use of antibiotics.Frequency of upper respiratory tract infections was ascertained by asking the following question, "How often does your child have an upper respiratory tract infection, with at least 2 of the following symptoms: cough, runny nose, fever?" Available responses were either never, 1-4 per year, 5-8 per year, 9-12 per year or >12 per year.

3.7.2 Wheezing

Wheeze was defined as a positive response to the question "Has your child ever had wheezing or whistling in the chest at any time in the past?" To augment this question a DVD of a preschool child wheezing was shown (Paediatric Respiratory Examination, OCB Media) and parents asked to report if their child's breathing had ever been like the child's on the video. Subsequent questions inquired about age of first wheezing, presence of wheezing in the last 12 months, number of attacks of wheezing in the last 12 months, parental assessment of whether their child has asthma, wheezing after exercsie or exertion, coughing at night without a cold, presence of wheezing without a cold and use of medications (B2 agonist inhalers, steroid inhalers, monteleukast and oral steroids.)

3.7.3 Rhinitis

Rhinitis was defined as a positive response to the question "Has your child ever had a problem with sneezing, or a runny, or blocked nose when he/she DID NOT have a cold or the flu?" Subsequent questions inquired about presence of these symptoms in the last 12 months, whether symptoms associated with itchy-watery eyes, which months of the year theses symptoms occured, parental reported diagnosis of hayfever and doctor diagnosis of allergic rhinitis.

3.7.4 Eczema

Eczema was defined as a positive response to the question, “Has your child ever had an itchy skin rash that was coming and going for at least six months?” Subsequent questions inquired about the presence of ezcema in the last 12 months, distribution of the rash, age of onset, sleep distrurbance, parental diagnosis of asthma and history of doctor diagnosed eczema.

3.7.5 Food allergy

Possible food allergy was defined as a positive response to the question “Has your child ever had a possible food allergy”. Subsequent questions enquired about possible food allergies to specific foods (egg, cow’s milk, peanut, tree nut, fish, seafood, soy, wheat, sesame, other) and whether the child had a doctor diagnosed food allergy.

3.7.6 Determinants of respiratory and atopic outcomes in childhood

Children were characterised by birth weight, gestation, admission to a neonatal unit at birth, immunisation status, presence of congenital abnormalities, presence of other health problems and use of regular medications.
The following genetic and environmental determinants of child health were assessed: ethnic background, age parents left full time education, number of adults and children in the household, attendance at nursery, number of older and younger siblings, exposure to household pets since birth, exposure to prenatal and postnatal tobacco smoke and history of doctor diagnosed eczema, rhinitis or asthma in the mother, father or siblings.

3.7.7 Determinants of child vitamin D status

The following determinants of child vitamin D dietary intake were assessed: any use of multivitamin and vitamin D supplements, mode of infant feeding, duration of breast feeding, age of weaning, observation of a restricted diet (vegan or vegetarian) and frequency of consumption of certain foods containing vitamin D (oily fish, margarine, eggs).
The following determinants of vitamin D production from sunlight were assessed: average length of time spent outdoors in daylight hours each day in the last month, use of sunscreen or clothing to protect from the sun oin sunny weather, length of time watching TV or using a computer each day, access to a garden, access to a terrace or balcony, parentally reported Fitzpatrick score of skin type.

3.7.8 Determinants of maternal vitamin D status

The following determinants of maternal vitamin D dietary intake were assessed: use of multivitamin and vitamin D supplements, observation of a restricted diet (vegan or vegetarian) and frequency of consumption of foods containing vitamin D (oily fish, margarine, eggs).
The following determinants of vitamin D production from sunlight were assessed: wearing concealed clothing (no concealed clothing vs all body covered except hands and face when outdoors vs all body covered including hands and face when outdoors), average length of time spent outdoors in daylight hours each day in the last month, use of sunscreen or clothing to protect from the sun in sunny weather, length of time

watching TV or using a computer each day, use of sunbed in the last year, Fitzpatrick score of skin type.

3.8 Follow up: Data collected by medical assessment

1. Height
2. Weight
3. Colorimetry (a measure of skin darkness)
4. SCORAD assessment
5. Respiratory resistance, impedance, reactance and resonant frequency were measured through a frequency range of 5Hz to 30Hz using impulse oscillometry
6. Bronchodilator response: Impulse oscillometry was performed before and after inhalation of 400micrograms of salbutamol
7. Exhaled nitric oxide using an offline technique
8. Skin prick tests for atopy: House dust mite, alternaria, cladosporum, cat, dog, grass pollen, silver birch, peanut, milk and egg

9. Analysis of peripheral blood mononuclear cells: flow cytometry and stimulation assays
10. Measurement of total IgE
11. Measurement of the eosinophil count
12. Measurement of the 25-OH-vitamin D level
13. Blood stored for DNA analysis
14. Maternal colorimetry
15. Maternal buccal swab for DNA

3.9 Electronic health record

Where consent was given, electronic health records were obtained from participants' primary care practitioners. Data regarding healthcare utilization and prescription costs were extracted from the records. A secondary analysis of the records was made by a consultant paediatric allergist (RB) as to whether individual children had a pattern of healthcare attendance and prescriptions supportive of a diagnosis of i. eczema ii. Recurrent wheezing (at least 2 episodes of acute illness with wheezing documented or suggestive clinical findings/prescription patterns documented) and iii. Food allergy.

3.10 Timing of data collection

The first child was recruited in July 2010. The last child was recruited in March 2011.

4 GENERAL ISSUES FOR STATISTICAL ANALYSIS

All analyses will be conducted two sided and significance interpreted at the 5% significance level.

4.1 Blinding of the statistical analysis

Statistical analysis will be completed using treatment codes A and B to designate assignment to prenatal vitamin D supplementation or no supplementation. Secondary analyses will be undertaken on the primary outcome measure using treatment codes C, D and E to designate assignment to bolus vitamin D, daily vitamin D or no treatment.

4.2 Analysis populations

4.2.1 Intent-to-treat population

The intention-to-treat (ITT) sample is defined for this trial as all participants randomized into the trial included in the intervention group to which they were randomised.

4.2.2 Available-case population

All participants for whom data are available for any given outcome measure.

4.2.3 Per protocol population

No per protocol analysis is planned for this trial.

4.2.4 Safety population

No specific safety population has been defined for this trial.

4.2.5 Other populations

N/A

4.3 Database

4.3.1 Description

The data are stored on a Microsoft Excel 2007 spreadsheet.

4.3.2 Data quality

Data from questionnaires and medical assessments were recorded onto paper. Each child had their own file. Each file was loaded onto an Epidata database which was exported to Excel.

Stephen Goldring and Heather Hanna (Research nurse) manually verified epidata files were correct by simultaneously reading through each file whilst checking the Epidata entry.

Stephen Goldring made a final check of all files in Epidata on 1/5/11 before converting the file to an excel speadsheet. He then added in data that had been recorded but not correctly coded for in the original Epidata check file. Stephen Goldring and Robert Boyle then range checked the data and reviewed data collection sheets for outliers prior to database closure.

4.3.3 Database freeze

The database will be completed and sent to Richard Hooper in May 2011, who will conduct or oversee data analysis. The database will be considered locked at this stage. This means that the database will be made read-only. If during the analysis unforeseen queries are generated they will be dealt with on a case-by-case basis. Any subsequent changes to the data can be made but will be recorded and reported.

4.4 Analysis software

The analysis will be carried out using Stata version 10.1.

4.5 Methods for withdrawals, loss to follow-up and missing Data

Available case analysis will be used, without imputation of missing data.

4.6 Method for handling centre effects

Non-applicable

4.7 Method for handling randomisation stratification or minimisation factors

Analysis will adjust for race as this was a stratification factor.

4.8 Method for handling clustering effects

N/A

4.9 Method for selecting other variables that will be adjusted for

We will control for variables in Table 1 which show imbalance – see section 6.

4.10 Multiple comparisons and multiplicity

There is only one primary analysis. There will be a number of secondary analyses but we will not make any adjustment for multiple testing as they all relate to hypotheses of prior interest

4.11 Method for handling non-adherence

Adherence was not formally documented in this trial, and will therefore not be taken into consideration in analyses.

4.12 Method for handling time-varying interventions

N/A

4.13 Method for handling outliers

Not to be excluded

4.14 Derived and computed variables

For complicated derivations/manipulation of data required please describe here, simpler derivations can be held in the analysis files.

Asthma predictive index(Castro-Rodriguez 2010) – 'loose' criteria
Defined as: Wheezing plus one of two major criteria, or two of three minor criteria

Major criteria
Q81. History of parent with doctor diagnosed asthma.
Q47. History of doctor diagnosed eczema.

Minor Criteria
Q39. History of doctor diagnosed allergic rhinitis?
Q24. History of wheezing without a cold.
Eosinophilia (≥4%)

Example text: All derived and computed variables will be documented in the analysis programmes.

5 DESCRIPTIVE ANALYSES

5.1 Participant flow

Participant throughput will be summarized in a CONSORT diagram.

5.2 Representativeness of sample

Should describe how you may look at whether your sample is representative of the population

5.3 Baseline comparability of randomised groups

5.3.1 Demographics

The following demographic data were collected by questionnaire at follow up. Key variables for inclusion in Table 1 and adjustment in statistical analyses (see 5.9) are highlighted:

Ethnic group of father
Ethnic group of mother
Age mother left full time education
Age father left full time education
Number of adults in the household
Number of children in the household
Whether child attends nursery, and from what age did they start
Number of older siblings
Number of younger siblings
Presence of household pets
Presence of household smokers
Maternal smoking during pregnancy
Family history of doctor diagnosed eczema/ asthma/ rhinitis (ie at least 1 parent with at least 1 disease)
Completed all immunisations

History child vitamin supplementation – Y/N
Mode of infant feeding – exclusively breast fed for at least 4 months – Y/N
Limited dietary history for the child
Sunlight questionnaire for the child

Child Fitzpatrick skin colour grade
History of current maternal vitamin supplementation
Limited dietary history for the mother
Sunlight questionnaire for the mother

The following data were collected in the original prenatal study
Baseline maternal vitamin D status
Cord 25-OH-vitamin D level

The following data were collected by assessment at follow up
Body mass index of the child
25-OH-vitamin D level in the child at time of assessment

Child's abdominal skin colorimeter reading
Mother's forearm skin colorimeter reading

5.3.2 Prior and concurrent medications
5.3.3 Baseline and screening conditions
5.3.4 Baseline medical history
5.3.5 Baseline physical exam
5.3.6 Cluster characteristics if cluster randomised
5.3.7 Characteristics of care providers where applicable

5.4 Comparison of losses to follow-up

5.5 Comparison of compliance to treatment and protocol

5.6 Emergency or accidental unblinding of randomised treatment

6 INTERIM ANALYSES AND SAFETY MONITORING ANALYSES

Non-applicable

6.1 Purpose of interim analyses

6.2 Monitoring plan

This should also include details of the planned number of interim analysis, timing of meetings during the trial, timing of data extraction prior to meeting, timing of meetings in relation to steering committee meetings. If safety data is being monitored differently to efficacy data the monitoring procedure should also be described here

6.3 Stopping rules

6.4 Measures taken to minimize bias

6.5 Adjustment for p-values

6.6 Interim analysis for sample size adjustment

7 ANALYSIS OF PRIMARY OUTCOME

Definition of outcome measure

Ever wheezing
Number of children with any wheezing episode in the first 3 years of life, defined as a positive response to the question
Q13. Has your child ever had wheezing or whistling in the chest at any time in the past?"

7.1 Descriptive statistics for outcome measure
7.2 Primary analysis
Analysis of binary outcomes will be by logistic regression adjusting for potential confounders (see section 6.3), and for continuous outcomes will be by analysis of variance, again adjusting for the factors/covariates described in section 6.3. In all cases the primary comparison will be between children randomized to 'any prenatal vitamin D supplementation' and the control group.

7.3 Assumption checks
Other analysis supporting the primary (inc. sensitivity analyses)
-Effect of bolus prenatal vitamin D versus no treatment on prevalence of 'Ever wheeze'
-Effect of daily prenatal vitamin D versus no treatment on prevalence of 'Ever wheeze'
-Analysis of effects of randomization on the primary outcome measure will be explored by repeating the analysis for those women with vitamin D deficiency at baseline (<25nmol/L) and for those with vitamin D insufficiency/deficiency at baseline (<50nmol/L).

8 ANALYSIS OF SECONDARY OUTCOMES

8.1 Definition of outcome measures

1. Wheezing related

c. Recurrent wheezing
Defined as ≥2 episodes of wheezing since birth. (Q17)

d. Wheezing episodes in the past 12 months
The percentage (%) of children with any wheezing episode in the last 12 months. Defined as a positive response to the question
Q16. Has your child had wheezing or whistling in the chest in the past 12 months?

e. Ever wheezing in children with and without a positive Asthma predictive index
Positive API (loose criteria) defined as wheezing plus one of two major criteria, or two of three minor criteria.
Major criteria Q81. History of parent with doctor diagnosed asthma.
Q47. History of doctor diagnosed eczema.

Minor Criteria Q39. History of doctor diagnosed allergic rhinitis?
Q24. History of wheezing without a cold.
Eosinophilia (≥4%)

f. Any prescription of inhaled bronchodilators
Defined as total number of children ever prescribed inhaled bronchodilators (Q25)

g. Physiological markers of lung function
For each child the mean of at least 2 acceptable readings for the following parameters measured by impulse oscillomery pre and post bronchodilator will be reported.
Resonant frequency (Fr) (Hz)
Area under the reactance curve (AX) (kPa/L)
Resistance at 10 and 20 Hz (R10 and R20) (k/Pa/(L/s)

h. Exhaled nitric oxide (ppb)
The median value of at least two offline measurements of exhaled nitric oxide.

i. Blinded consultant paediatrician's assessment of recurrent wheeze using primary care electronic health record

2. Eczema and atopy related

a. Eczema ever
Number of children with eczema in the first 3 years of life, defined as a positive response to the question:

Has your child ever had an itchy skin rash that was coming and going for at least 6 months (ie Q.40)?

b. Eczema in the past 12 months
Number of children with eczema in the last 12 months, defined as a positive response to the questions (ISAAC definition):
Q41. Has your child had this itchy rash at any time in the past 12 months? AND
Q42. Has this itchy rash, at any time, affected any of the following places: The folds of the elbows, behind the knees, In front of the ankles, under the buttocks, or around the neck, ears or eyes?

c. Atopy
Atopy defined as % of children with positive skin prick test, defined as a positive response to at least one allergen, and a positive response is defined as a wheal diameter ≥3 mm in the presence of appropriate responses to the negative and positive control.

d. Total IgE
Measured as a continuous variable

e. Allergic rhinitis
Defined as above (3.7.3)

f. Food allergy (doctor diagnosed)
Defined as above (3.7.5)

j. Blinded consultant paediatrician's assessment of eczema using primary care electronic health record

k. Blinded consultant paediatrician's assessment of food allergy using primary care electronic health record

3. Infection related

a. Frequency of upper respiratory tract infections per year. Defined as:
Q12. How often does your child have an upper respiratory tract infection, with at least 2 of the following symptoms: cough, runny nose, fever? ('Never', '1-4 per year', '5-8 per year', '9-12 per year', '>12 per year', To be dichotomized as ≤4 or >4.

b. Frequency of lower respiratory tract infections since birth.
Defined as a positive response to the question:Q9. Since your child was born have you ever been told by a health professional that your child has bronchiolitis, bronchitis, croup, pneumonia or a chest infection?

4. Healthcare costs

a. Total healthcare costs to the NHS, estimated from GP electronic health record
Will be calculated from analysis of primary care health records for each child..

b. Total healthcare attendance costs related to respiratory illness
Will be calculated using standardized health economic data

c. Total healthcare attendance costs related to allergic illness
Will be calculated using standardized health economic data

d. Total healthcare costs to the NHS during first year of life

8.2 Descriptive statistics for outcome measure

8.3 Secondary analysis

Analysis of binary outcomes will be by logistic regression adjusting for potential confounders (see section 6.3), and for continuous outcomes will be by analysis of variance, again adjusting for the factors/covariates described in section 6.3.

8.4 Assumption checks

8.5 Other analysis supporting the secondary (inc. sensitivity analyses)

Effect of bolus prenatal vitamin D versus no treatment on prevalence of 'Ever wheeze'

Effect of daily prenatal vitamin D versus no treatment on prevalence of 'Ever wheeze'

Analysis of effects of randomization on the primary outcome measure will be explored by repeating the analysis for those children whose mothers had vitamin D deficiency at enrolment (<25nmol/L) and for those with vitamin D insufficiency/deficiency at enrolment (<50nmol/L).

<u>Other analyses which will be performed as part of this trial, but are not part of this analysis plan for clinical outcomes, are:</u>

1. A more detailed analysis of health economic outcomes will be undertaken and reported separately, to investigate whether prenatal vitamin D supplementation has an effect on specific types of healthcare utilization for specific conditions at specific ages.

2. A detailed analysis of immune responses at age 3 years will be undertaken, to investigate the effects of prenatal vitamin D supplementation on immune development. These include circulating regulatory T cell numbers, dendritic cell numbers, phenotype and ILT-3 expression, and whole blood response to 24 hour culture with mitogen, allergen (ovalbumin), infective agent (tetanus toxoid, rhinovirus RV-16), pathogen associated molecular pattern (TLR 1-9) measured as Th1, Th2, proinflammatory cytokine or IFN-alpha response. This analysis will use the same 2 dichotomised groups (vitamin D versus no vitamin D) as the clinical dataset, with exploratory analyses of bolus vitamin D and daily vitamin D treatment and cord blood vitamin D level.

Important variables for inclusion in Table 1 and/or in adjusted analyses for these outcomes are mode of delivery, maternal age, gestational age, birth weight, number of prior pregnancies, smoking during pregnancy, current household smoker, current infection at time of blood test, number of children in household, frequency of upper respiratory tract infections, current vitamin D level, vaccination status (to tetanus), BMI z-score, sex, presence of atopy, total IgE level.

3. An analysis of determinants of vitamin D status in mothers (at baseline) and children will be undertaken using questionnaire data and colorimeter readings.

4. An analysis of the relationship between current vitamin D status and immune response to microbial and non-microbial stimuli will be undertaken using results from blood assays at 3 years.

9 SAFETY AND TOLERABILITY ANALYSES

9.1 Drug exposure

Describe exposure to drug in terms of compliance

9.2 Adverse events

Premature delivery (<36 weeks gestation)
Low birth weight (<2.5 kg)
Neonatal unit admission
Death – stillbirth/neonatal death/postneonatal death, and causes
Congenital abnormalities
Chronic non-atopic health condition in child (Q8)
Haemaglobin, MCV, white cell count, neutrophil count, lymphocyte count, platelet count at age 3
Height at 3 years (z-score)
Weight at 3 years (z-score)
Body mass index at 3 years (z-score)

9.3 All adverse events

9.4 Adverse events leading to withdrawal

9.5 Serious adverse events

9.6 Clinical laboratory evaluations

10 SUBGROUP ANALYSES

10.1 Definition of outcome measure

10.2 Definition of subgroups

10.3 Sample size justification for the subgroup analysis

10.4 Descriptive analysis for subgroups

10.5 Method of analysis

11 AMENDMENTS TO VERSION X

Any amendments to the previously agreed analysis plan should be detailed here, including the rational for changes.

12 REFERENCES

13 APPENDIX

Where scoring of questionnaires is required for the analysis they should be attached here

Camargo, C. A., Jr., S. L. Rifas-Shiman, et al. (2007). "Maternal intake of vitamin D during pregnancy and risk of recurrent wheeze in children at 3 y of age." clinical study Am J Clin Nutr **85**(3): 788-795.

Castro-Rodriguez, J. A. (2010). "The Asthma Predictive Index: A very useful tool for predicting asthma in young children." Journal of Allergy and Clinical Immunology **126**(2): 212-216.

Martinez, F. D., A. L. Wright, et al. (1995). "Asthma and wheezing in the first six years of life. The Group Health Medical Associates." N Engl J Med **332**(3): 133-138.

Yu, C. K., L. Sykes, et al. (2009). "Vitamin D deficiency and supplementation during pregnancy." Clin Endocrinol (Oxf) **70**(5): 685-690.
